# Unraveling the Structural Variations of Early-Stage Mycosis Fungoides—CD3 Based Purification and Third Generation Sequencing as Novel Tools for the Genomic Landscape in CTCL

**DOI:** 10.3390/cancers14184466

**Published:** 2022-09-14

**Authors:** Carsten Hain, Rudolf Stadler, Jörn Kalinowski

**Affiliations:** 1 Center for Biotechnology (CeBiTec), Bielefeld University, 33615 Bielefeld, Germany; 2 University Clinic for Dermatology, Johannes Wesling Medical Centre, UKRUB, University of Bochum, 32429 Minden, Germany

**Keywords:** cutaneous T-cell lymphoma, mycosis fungoides, enrichment, sequencing, nanopore, copy-number variation, structural variation

## Abstract

**Simple Summary:**

Mycosis fungoides is the most common cutaneous T-cell lymphoma, but knowledge of the genetic alterations, particularly of the early stages, is limited. A major problem is that biopsies from early stages contain few tumor cells and many “healthy” skin cells, making accurate analysis of tumor cells difficult. Here, we demonstrate a workflow to enrich tumor cells and thereby obtain better results for mutation detection, especially for deletions and amplifications. For the same sample, we also demonstrate the advantages of long-read sequencing for a more comprehensive elucidation of genetic alterations in early stages of mycosis fungoides.

**Abstract:**

Mycosis fungoides (MF) is the most common cutaneous T-cell lymphoma (CTCL). At present, knowledge of genetic changes in early-stage MF is insufficient. Additionally, low tumor cell fraction renders calling of copy-number variations as the predominant mutations in MF challenging, thereby impeding further investigations. We show that enrichment of T cells from a biopsy of a stage I MF patient greatly increases tumor fraction. This improvement enables accurate calling of recurrent MF copy-number variants such as *ARID1A* and *CDKN2A* deletion and *STAT5* amplification, undetected in the unprocessed biopsy. Furthermore, we demonstrate that application of long-read nanopore sequencing is especially useful for the structural variant rich CTCL. We detect the structural variants underlying recurrent MF copy-number variants and show phasing of multiple breakpoints into complex structural variant haplotypes. Additionally, we record multiple occurrences of templated insertion structural variants in this sample. Taken together, this study suggests a workflow to make the early stages of MF accessible for genetic analysis, and indicates long-read sequencing as a major tool for genetic analysis for MF.

## 1. Introduction

Mycosis fungoides (MF) is the most common form of cutaneous T-cell lymphoma (CTCL) and manifests as inflammatory lesions on the skin [1,2]. Early-stage MF often shows fixed skin lesions, called patches. In advanced stages of the disease, partly concurrent development of more infiltrated plaques or tumors are observed [1,3]. Furthermore, involvement of lymph nodes or visceral organs is possible [4,5,6]. Disease progression is patient-specific, with 20–30% progressing into an aggressive advanced stage disease with life expectancies between 1.5 and 4 years. The rest remains in an indolent early-stage disease with normal life expectancy [4,7,8]. A prognosis about the course of the disease is possible by clinical and histological features via the “Cutaneous Lymphoma International Prognostic index” (CLIPi) score [9,10] or via tumor clone frequency by T-cell receptor sequencing [8]. This prognosis is crucial as indolent and aggressive forms of MF require different forms of therapy: ranging in its extremes from a watch-and-wait strategy for indolent disease, to systemic therapy in the advanced form [3,11,12,13] with new targeted therapies such as brentuximab vedotin [14] or mogamulizumab [15].

Currently, a prognosis linking somatic mutations to disease progression and potential success of treatments, as (partly) present in other cancers [16,17,18], does not exist for MF. One reason why there is little correlation between genetics and possible prognosis of disease progression in MF is the lack of sufficient genetics data on the different stages of MF. While CTCL as a whole is a disease with a large genomic data source and more than 250 sequenced samples [19,20,21,22,23,24,25,26,27,28,29,30], MF datasets are a minority, accounting for only one fifth of all samples. Moreover, MF samples mostly stem from tumor-stage patients, often with a long history of different treatments. This makes it difficult to distinguish between driver mutations that initially determine disease progression, and secondary mutations that have less impact on early tumor development.

One major obstacle in analyzing early-stage MF skin lesions is the general low number of malignant cells in skin biopsies. In MF, atypical cells are found in variable numbers dependent on the disease stage [31]. During early stage, malignant cells are in the minority [32,33] and the fraction of malignant cells increases in well-established lesions in advanced stages [34]. In cases of Sézary syndrome (SS), the most often sequenced form of CTCL, tumor cells are enriched to generally high purities, as CD4+ cells from peripheral blood mononuclear cells [21,23,25,26,27] or CD3+CD26-CD14-CD8-CD19- cells from blood [20]. For MF, either whole biopsies [19,22], or microdissection of tumor cells after HE- [35] or CD3-staining [8] are used.

Most studies on CTCL genomics were done using whole-exome sequencing (WXS). This technique enables cost-effective high sequencing depth for coding sequences and shows high-sensitivity for single nucleotide variants (SNV) with limit-of-detection around 5% [36,37,38]. In contrast, WXS shows poorer sensitivity for the detection of copy-number variants (CNV) [39,40,41,42], a problem that is magnified in cancer samples with increased heterogeneity and decreasing tumor fraction [43]. As CNVs are very common and important in MF [19,22,28], careful attention on CNV calling performance in MF samples is necessary. Another disadvantage of WXS is that it only finds amplifications and deletions and not the causative structural variants (SV) leading to these CNVs. Thus, for a complete detection of SV, whole-genome sequencing (WGS) is needed.

Recent improvements in third generation sequencing makes these methods especially interesting for SV calling. Long-read lengths enable high-confidence mapping even in low complexity or repeating regions [44,45]. They allow phasing of multiple SV breakpoints into one haplotype [46] and enable the direct identification of complex SVs [47,48]. Current problems such as higher per-base error-rate are steadily reduced by new (bio)chemistry and bioinformatic tools [49].

## 2. Materials and Methods

### 2.1. Access to Restricted Data and Patient Consent

For the analysis of tumor fraction in CTCL, WGS, and WXS data of 76 SS patients from 3 studies [20,23,25], 11 MF patients from 2 studies [22,23], and 9 samples from this work were aggregated. All original studies stated that all patients gave written informed consent. The data from Choi et al. 2015 [20] and McGirt et al. 2015 [22] was publicly available on SRA. The data from Ungewickell et al. 2015 [23] and Wang et al. 2015 [25] is hosted on dbGaP with consent groups of Health/Medical/Biomedical or General Research Use, respectively. We applied successfully for both controlled datasets, clearly stating our intentions of combining multiple cohorts of patients from different studies.

### 2.2. Data Accession

Access to the controlled datasets phs000913 [23] and phs000725 [25] was requested via dbGaP. Paired-end FASTQ data from these datasets as well as the SRA datasets SRP058948 [20] and SRP059214 [22] was downloaded with the SRA toolkit 2.8.2. For each individual, WGS or WXS data for a tumor and a normal sample was downloaded. Sample SRR2046920 was an exception, since only tumor data were available.

### 2.3. Sample Collection

This study’s subject is a 79-year-old male (at the time of sample collection) with IB stage MF (T1b N0 M0 B1) of the CD4 phenotype. Written consent was obtained from the patient. A spindle biopsy from a skin lesion was taken, from which 8 × 5 mm punch biopsies were obtained and used for further analysis.

### 2.4. Tissue Disruption, Enrichment of CD3+ and CD4+ Cells, and DNA Isolation

In two aliquots, four 5 mm punch biopsies were disrupted into single cells using the human Whole Skin Dissociation Kit (Miltenyi Biotec, Bergisch Gladbach, Germany) according to the manufacturer’s protocol, with a 3 h incubation at 37 °C in each of two aliquots. Enzyme P was not added to the aliquot intended for CD4+ isolation. A small aliquot, suitable for DNA isolation, was taken from the disrupted tissue with Enzyme P added. From the remaining material, CD3+ or CD4+ cells were captured using human CD3 or CD4 MicroBeads (Miltenyi Biotec, Bergisch Gladbach, Germany) on MS Columns (Miltenyi Biotec, Bergisch Gladbach, Germany). From single cells, as well as CD3+ or CD4+ enriched cells, DNA was isolated using the DNeasy Blood & Tissue Kit (Qiagen, Hilden, Germany).

### 2.5. Whole-Exome Sequencing

For whole-exome sequencing, 150 ng of sample DNA was sheared to 300 bp length using Covaris microTUBEs (Covaris, Woburn, MA, USA). Library construction was carried out with the KAPA HyperPrep Kit (Roche, Basel, Switzerland) with IDT xGEN^®^ Dual Index UMI Adapters (IDT, Coralville, IA, USA) and four PCR cycles. Afterwards, exon capturing using the IDT xGEN^®^ Exom Research Panel v1.0 (IDT, Coralville, IA, USA) was carried out according to manufacturer’s protocol. The library was size-selected to 350–560 bp using the BluePippin device (Sage Science, Beverly, MA, USA), quantified using Qubit dsDNA HS (ThermoFisher, Waltham, MA, USA), evaluated via capillary electrophoresis using Bioanalyzer (Agilent, Santa Clara, CA, USA), and ultimately sequenced on Illumina NextSeq 500 sequencer with 2 × 150 bp read length.

### 2.6. Short-Read Data Processing and Variant Detection

Short-read data processing and detection of somatic single nucleotide variants and copy-number variants was carried out as previously described [50]. In addition to the gatk-somatic-cnvs pipeline from GATK 4.1.4.0 [51], CNV calling was performed with CNVkit 0.9.10 [52] or CONTRA 2.0.8 [53] using standard parameters.

### 2.7. Long-Read Whole-Genome Sequencing and Data Processing

From the CD3+ sample, 100 ng DNA was subjected to library construction using the SQK-PBK004 kit and sequenced on the GridION sequencer with R9.4.1 flow cells (Oxford Nanopore Technologies, Oxford, UK). Base calling was performed with guppy 5.1.13 and the super-accuracy model. Reads were trimmed with Porechop 0.2.4 [54] and mapped to GRCh38 with minimap2 2.17 [55] using the -R, -Y, and the –MD parameters. Coverage analysis was performed by counting bases in 50 kb intervals using samtools and visualizing the results using a custom script. Structural variants were called using Sniffles 2.0.5 [56,57] with the –non-germline, –output-rnames, and –tandem-repeats parameters. The called SVs were filtered using a custom script available on GitHub (https://github.com/carstenhain/sv-analysis, accessed on 6 August 2022). SVs were visualized using Circos 0.69 [58] and custom python scripts.

### 2.8. Long-Read Targeted Sequencing

A total of 1 µg of DNA from the unprocessed spindle biopsy was subjected to library construction using the SQK-LSK109 kit and sequenced on the GridION sequencer with R9.4.1 flow cells (Oxford Nanopore Technologies, Oxford, UK). The adaptive sampling option was enabled in MinKNOW 20.10.6. For targeting, a bed-file and the human genome GRCh38 were used. The intervals were constructed manually, consisting of individual SV breakpoints with 15 kb padding as well as larger regions, e.g., the complete chr9p region. In total, 112 regions comprising 112 Mb were targeted with this approach (Table S2). Base calling was done with guppy 5.1.13 and the super-accuracy model. Reads were trimmed with Porechop 0.2.4 [54] and mapped to GRCh38 with minimap2 2.17 [55] using the -R, -Y, and the –MD parameters. SNP calling was performed with longshot 0.4.1 [59], SNPs were filtered for QUAL > 100, and reads were phased with WhatsHap [60].

### 2.9. Detection of RAG or Microhomology Sites at SV Breakpoints

Analysis of RAG heptamers was carried out as described [20]. For microhomology detection, the sequences 12 bp around SV breakpoints were extracted and pairwise aligned using a custom script. The best alignment (in bp length) was used for further analysis. For comparison with RAG and microhomology occurrence in a random background, a random set of 100 SVs was generated and subjected to the same analysis steps as the actual SVs.

### 2.10. Detection of Templated Insertions

During SV filtering, all supporting reads from single SVs were assembled using lamassemble [61]. SV assemblies were mapped to GRCh38 with minimap2, and assemblies mapping to more than two regions in the genome were flagged and inspected manually.

### 2.11. Validation of SV Breakpoint Sequences Using Sanger Sequencing

Selected SVs detected by nanopore whole-genome sequencing were validated by Sanger sequencing. PCR products for sequencing were prepared using individual primers (Table S1), cleaned up with ExoSAP-IT™ PCR Product Cleanup Reagent (ThermoFisher, Waltham, MA, USA), and sequenced using one PCR primer. Results were mapped and analyzed with Geneious 2020 (Geneious, Auckland, New Zealand).

### 2.12. Assembly and Identification of T-Cell Receptor Gamma Alleles

Reads spanning the two most common V/J combinations of the T-cell receptor gamma (*TRG*) were extracted and assembled using lamassemble [61]. The assembly was annotated with MiXCR [62]. For further analysis, reads were mapped to the assemblies and reads with multiple errors in the complementary determining region (CDR3) were removed. The assemblies were mapped to GRCh38 using minimap2 2.17 [55].

## 3. Results and Discussion

### 3.1. Early-Stage Mycosis Fungoides Biopsies Show Low Tumor Purity

**Figure 1:**
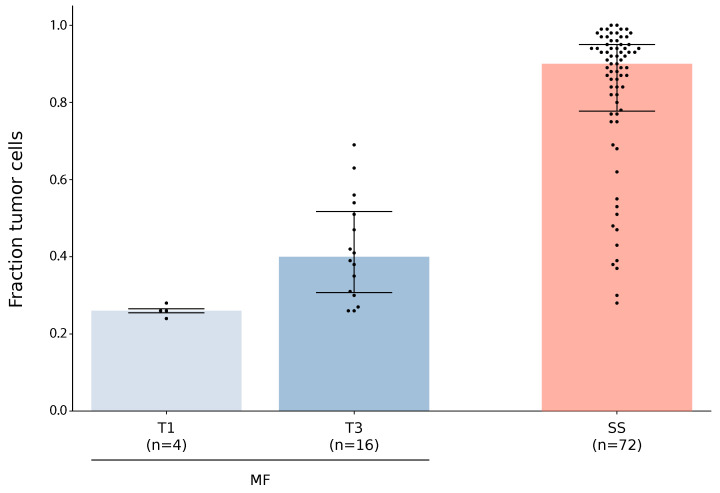
Fraction of tumor cells in samples from different forms and stages of CTCL. WXS or WGS data from this work or from published studies [20,22,23,25] were evaluated for tumor cell fraction using ABSOLUTE [63]. The samples are grouped and classified, according to patient information, into MF with tumor stage 1 or 3, or SS. For each class, sample size, median, and first and third quantile are depicted by horizontal bars.

We sought to quantify the fraction of tumor cells in sequenced samples from different forms and stages of CTCL. For this analysis, we gathered CTCL whole-exome (WXS) or whole-genome sequencing data. After quality control, 72 SS [20,23,25] and 4 tumor stage (T) 1 MF (this work) as well as 16 tumor stage 3 MF [22,23] (and this work) remained. For each sample, somatic SNVs and CNVs were called and tumor cell fraction was calculated with ABSOLUTE [63] (Figure 1). As expected, the T1 MF samples showed the lowest tumor cell fraction (median: 0.26; range: 0.24–0.28) followed by the T3 MF samples (median: 0.4; range: 0.26–0.69), while the SS samples contained the highest fraction of tumor cells (median: 0.9; range: 0.28–1.0). Individual outliers, such as T3 MF samples with more than 60% tumor cells, were observed. Although the gathered dataset is small, especially for early-stage MF, we believe that our results indicate a general trend: that tumor fraction in early-stage MF is lower than in later stages. This is coherent with previous studies, showing that malignant cells in MF lesions are in the minority [32] in early stages and the proportion of malignant cells increases in established lesions [34]. However, the trend is strongly influenced by other effects such as patient-specific variance of the tumor fraction or sampling bias (e.g., biopsy size and position relative to the patch). In general, low tumor fraction in early-stage MF poses a major obstacle for high-confidence analysis of somatic mutations in this sample group. To overcome this issue, a bias-free enrichment of tumor cells from biopsies before molecular analysis is necessary.

### 3.2. Tumor Cell Enrichment Leads to Increased Sensitivity for Copy-Number Variation Detection

We aimed to analyze one early-stage MF sample with special interest to high-confidence and -sensitivity CNV calling. Furthermore, we proposed that enrichment of T cells via CD3 or CD4 and the associated depletion of the surrounding cell types, such as keratinocytes, increases the tumor content of the sample.

Eight fresh 5 mm punch biopsies of an MF patient with proven T-cell clonality in stage IB (Figure S1) were randomly split into two batches before dissecting each batch into single cells. From the batch designated for CD3+ enrichment, one aliquot of cells was kept as the unprocessed sample. CD3+ cells were isolated from the rest using CD3 MicroBeads. From the other batch, CD4+ cells were isolated using CD4 MicroBeads. A punch biopsy of uninvolved skin was used as a healthy control. From all four samples, DNA was isolated and subjected to whole-exome sequencing. The unprocessed and enriched tumor samples were sequenced to a depth of 192×, 170×, and, 149×, respectively. Somatic variants, SNVs and CNVs, were called and absolute copy-numbers as well as tumor fraction were analyzed using ABSOLUTE [63].

The fraction of tumor cells in the unprocessed biopsy was 0.24, 0.58 in the CD3+, and 0.45 in the CD4+ enriched sample. These results depict an enrichment of tumor cells by the factor of 2.42 in the CD3+ and by 1.88 in the CD4+ sample. The increased tumor fraction in the CD3+ samples compared to the CD4+ sample is unexpected as MF samples, including this patient, are mostly CD3+CD4+CD8- [64]. We assume that the difference lies in the experimental procedure, especially a less rigorous tissue disruption in the CD4 batch due to the sensitivity of CD4 towards one of the normally used, but in this case omitted, protease. Further experimental optimization might resolve this problem, leading to a more complete tissue lysis and efficient capturing of CD4+ cells. Additionally, imbalances in the tumor fraction between both starting batches are possible. The seemingly more robust purification of malignant MF cells via CD3 should work well for most cases as the ratio of CD4:CD8 cell in MF is consistently above 1 [65,66], thus, the larger fraction of CD3+ cells should be CD4+ as well. Furthermore, the purification via CD3 has the additional advantage in capturing the malignant cells in rare CD4-CD8- MF cases, which are mostly CD3+ [67]. In the future, a more sophisticated antigen combination and FACS purification might lead to an even higher enrichment of malignant cells from individual biopsies. However, careful consideration of the antigens presented by the malignant cells is vital for successful enrichment. As MF cells are known to lose pan T-cell antigens [64], careful immunohistochemistry is necessary prior to enrichment to detect (subclonal) loss of the enrichment antigen. In the case of subclonal antigen loss, only a subpopulation of malignant cells would be enriched, and therefore the enriched cells would not represent the entirety of malignant cells in the tumor. Besides immunohistochemistry, T-cell receptor (TCR) sequencing is also crucial to elucidate the clonal architecture of the lesion. In MF, there are cases with monoclonal TCR but also cases with oligoclonal TCR, the latter strongly indicating multiple subclonal populations in the lesion [28]. Additionally, TCR sequencing can be used to track complete enrichment of all subpopulations of malignant cells by sequencing prior and after enrichment and comparing the results.

**Figure 2:**
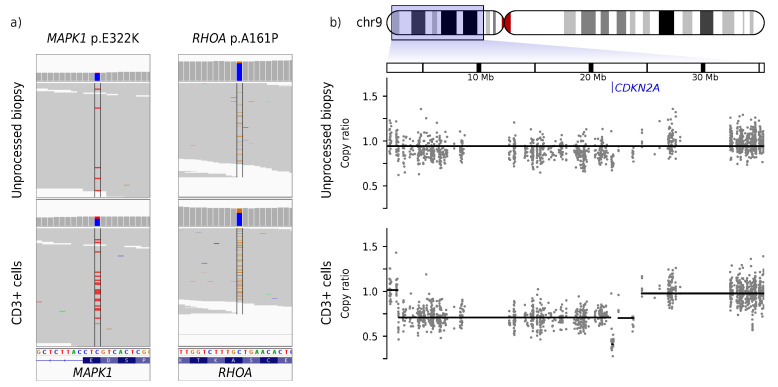
Somatic variant calling in an early-stage MF skin biopsy without (top) and with (bottom) enrichment of tumor cells. One skin lesion of a patient with tumor stage 1 MF was biopsied. One half (unprocessed biopsy) was used without further steps. The other half of the material was used for enrichment of CD3-positive cells (CD3+ cells). Afterwards, both halves were used for DNA isolation, WXS, and calling of somatic SNVs and CNVs. (**a**) Raw data for the somatic SNVs *MAPK1* p.E322K and *RHOA* p.A161P where colored strips indicate variant reads diverging from the genome reference. (**b**) Raw and processed data for a deletion on chr9 leading to homozygous loss of *CDKN2A*. The gray dots show the copy-ratio (denoised and normalized read depth of individual exons) while the black lines depict the automatic CNV call. A copy-ratio of 1 indicates a neutral copy-number of 2 on autosomes. The genomic position of *CDKN2A* is marked in blue.

Analysis of the somatic mutations revealed 762 SNVs in the CD3+ enriched sample. Further classification of SNVs with mutationTimeR [68] resulted in 737 clonal and 25 subclonal SNVs, corresponding to a clonal mutation rate of 14.5 mutations per Mb. Over 85% of clonal SNVs were C>T transitions. Of the 737 clonal SNVs in the CD3+ sample, 736 (99.8%) were found in the CD4+ and 716 (97.2%) were found in the unprocessed sample. Known oncogenic mutations such as *MAPK1* p.E322K and *RHOA* p.A161P were detected in all samples, albeit with 3.37× or 2.76× higher frequency in the CD3+ sample than in the unprocessed sample, respectively (Figure 2a). The gain-of-function *RHOA* p.A161P mutation [69] is similar to the mutation occurrence in other CTCL cases outside of the *RHOA* p.G17 hotspot commonly mutated in other cancers and lymphoma [70]. The gain-of-function *MAPK1* p.E322K mutation [71] is found exclusively in MF and not in SS [70].

In the CD3+ sample, 7.16% of the genome is hetero- and 0.03% homozygously deleted, while 10.28% of the genome is amplified. A total of 69 amplified or deleted segments were called. Similar results (60 amplified or deleted segments, visual agreement between both samples) were observed for the CD4+ sample (Figures S2 and S3). In the unprocessed sample, CNV calling classified 8.7% of the genome as deleted and 10.08% as amplified. Only 14 amplified or deleted segments were found. A clear distinction between homo- and heterozygous deletions was also not possible. CNV analysis using other CNV callers such as CONTRA [53] or CNVkit [52] yielded more noisy results (Figure S4).

In general, the unprocessed sample showed a much less sensitive CNV calling than the CD3+ sample. Exemplary and of special interest is the CNV on chr9 (Figure 2b). CNV calling in the CD3+ sample clearly shows two different levels of deletions: a large single-deleted region from 2.8 to 22.4 Mb and a small double-deleted region from 21.8 to 22 Mb. The tumor suppressor gene *CDKN2A*, which is located in the double-deleted segment on chr9:21.9 Mb, is thus homozygously deleted in this patient. CNV calling in the unprocessed sample cannot resolve these deletions and proposes one large weakly deleted region from chr9:0-64 Mb.

Insensitive CNV calling, especially for chromosomal segments considerably smaller than a chromosomal arm, is observed in the unprocessed sample at numerous locations. This includes failure to detect *STAT3/5* amplification and inaccurate detection of an *ARID1A* deletion (Figure S5).

A detailed analysis of this study’s patients showed multiple mutations recurrent for CTCL and partly specific for MF, including the *RHOA* and *MAPK1* SNVs as well as *CDKN2A* deletion, *STAT3/5* amplification, *ARID1A* deletion and chr7 trisomy [19,20,70,72]. The overlap in called SNVs between the unprocessed and the tumor enriched samples was very high, and especially the oncogenic mutations were called confidently in all samples.

This result is expected as WXS, without additional modifications, has a limit of detection at variant allele frequencies of 5–10% for SNVs [36,37,38]. In contrast, CNV calling in WXS is a difficult problem even in germline samples with presumable completely clonal CNVs [40]. CNV calling performs better in short-read WGS, but even there, little overlap between existing tools is reported [40,41], and an allele fraction of 20% is considered the minimum for accurate SV calling [43].

In tumor samples, this problem is aggravated due to lower fractions of tumor cells containing the CNVs, resulting in weaker signals of individual CNVs in the WXS data. In this concrete example, the signal-to-noise ratio of the unprocessed MF biopsy of single allele amplifications or deletions are close to 1 (1.12 and 1.07, respectively), while the signal-to-noise ratio of the CD3+ sample is 2.5 (2.6 and 2.55, respectively) (Figure S6). This difference in signal-to-noise ratio is the major reason for the insensitive CNV calling in the unprocessed sample. For large CNVs such as the trisomy 7 or the general mention that chr9p is affected by some kind of deletion, this dataset is sufficient. However, for a more detailed analysis of the individual CNVs, a more precise resolution of the CNV breakpoints and a better classification of how many alleles are deleted or amplified is crucial.

This can be illustrated using two examples:

The *STAT3/5* gain on chr17q is the most recurrent amplification in CTCL and is present in 60% of SS cases [70]. In SS [24,26,73] and myeloid neoplasms [74], this CNV is often caused by a chr17 isochromosome, leading to a simultaneous loss of 17p including *TP53*. In this sample, the *STAT3/5* genes are presumably amplified as a 466 kb segment, separate from another CNV, leading to amplification of the distal part of chr17q (starting from 43.5 Mb). This result indicates that the *STAT3/5* amplification in this sample is not due to an isochromosome or a large-scale amplification, but rather due to a smaller amplification. This might indicate another mutation mechanism than the one leading to isochromosomes [75].

Secondly, the homozygous *CDKN2A* deletion is detected in high resolution only in the CD3+ sample. In the unprocessed sample, the distinction between hetero- and homozygously deleted segments is not possible. The distinction between homo- and heterozygous deletion is important for the correct interpretation/prediction of the effect of the mutation. In case of haploinsufficient genes such as *CDKN2A* [76], loss of one allele already leads to a phenotypic change. Homozygous deletions lead to lower mRNA levels than heterozygous deletions for *CDKN2A* [77,78], indicating an even stronger effect. In *CDKN2A* and other tumor suppressors, a homozygous deletion—or an otherwise homozygous loss by a combination of deletion, SNV, or promoter methylation—leads to more severe disease with decreased overall survival [79,80,81]. However, in certain cases, stark differences in phenotype between hetero- and homozygously deleted/mutated genes are observed [82].

Taken together, only sufficiently large fractions of tumor cells enable analysis of the correct location and state of individual CNVs. This is an extremely important point for further research in MF as a whole, as CTCL does not show highly recurrent SNVs but strong recurrence in CNVs, highlighting the great importance of CNVs in CTCL [20]. This fits with our previous analysis suggesting that CNVs are among the earliest mutations in the pathogenesis of SS [50]. For the ongoing endeavor to build genomic knowledge of the currently underrepresented early-stage MF and potentially discern progressing from indolent forms, the following points have to be considered. In early MF pathogenesis, CNVs might also be the main unifying mutations, and low tumor fraction, which is present in early MF lesions, will hinder accurate and confident CNV analysis, thus hiding the main genetic change in MF. Therefore, careful and preferably bias-free enrichment of tumor cells above a certain threshold is mandatory to achieve high-quality data needed to build a complete picture of genetic changes in early MF. In this context, it is important to consider that enrichment of tumor cells does not capture the original (genetic) composition of the tissue, including the microenvironment. It makes a compromise to allow the detection of genetic changes in the tumor cells with increased confidence, on the expense of missing surrounding mutations. If changes in the tumor microenvironment are of special interest, other techniques such as single-cell RNA sequencing or spatial transcriptomics are better suited.

### 3.3. Nanopore Sequencing Reveals the Structural Variants Underlying Classical MF CNVs

WXS detects amplifications or deletions but does not locate the breakpoints of the causative structural variants (SVs) leading to these CNVs. As such, the exact class of SVs and possibly the biological mechanism leading to these SVs is not resolvable with WXS. For SV analysis, third generation long-read sequencing shows increased performance, especially in repeating or low-complexity regions [45], and enables direct identification of complex SVs consisting of multiple translocations.

To elucidate the SVs leading to the observed CNVs, we used nanopore long-read genome sequencing on the CD3+ sample. Specifically, we utilized the PCR barcoding kit and generated 31.7 million reads with a mean length of 2.1 kb, resulting in a 22.2× mean genome coverage. The application of a PCR kit was a compromise due to low sample DNA quantities. Sequencing of native long fragments enables more confident SV calling [56] and simultaneous analysis of the epigenome [83].

Structural variants were called with Sniffles2 [56,57] and filtered using a custom script. In the following, only SVs longer than 5000 bp or inter-chromosomal translocations are considered, thereby removing three quarter of germline SVs (Figure S7). Obviously, a correct tumor-normal-approach for removal of germline SV would be preferable and would enable correct identification of smaller somatic SVs. Under these criteria, 167 SVs (93 deletions, 16 inversions, 10 duplications, and 48 inter-chromosomal translocations) were found.

**Figure 3:**
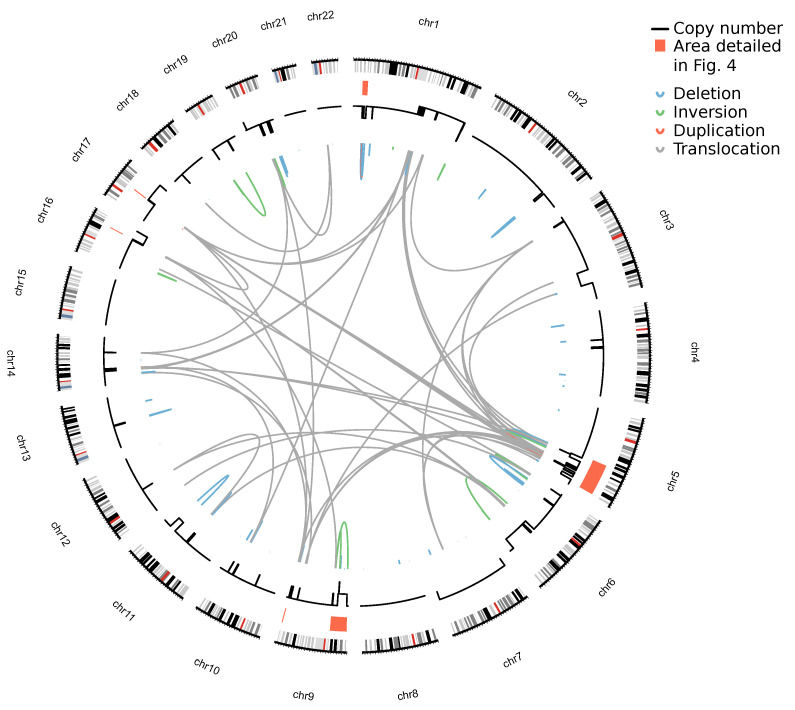
Structural variations in a early-stage MF sample detected by nanopore sequencing. Plotting of the copy-number landscape as derived from the WXS (black lines, outer ring) and the SVs detected by nanopore sequencing (colored lines, inner ring). Coloring of individual SVs indicates the SV class. The height of SV curves is proportional to the SV size. The areas marked in red are elucidated in Figure 4.

Combined plotting of SVs and CNVs yields an insight into the genomic architecture of the sample (Figure 3). In most cases, a breakpoint is located at (or close to) the edge between two genomic segments of different copy-number. These breakpoints are often clustered, as exemplified by the dense clusters on chr1, chr9, and chr17.

**Figure 4:**
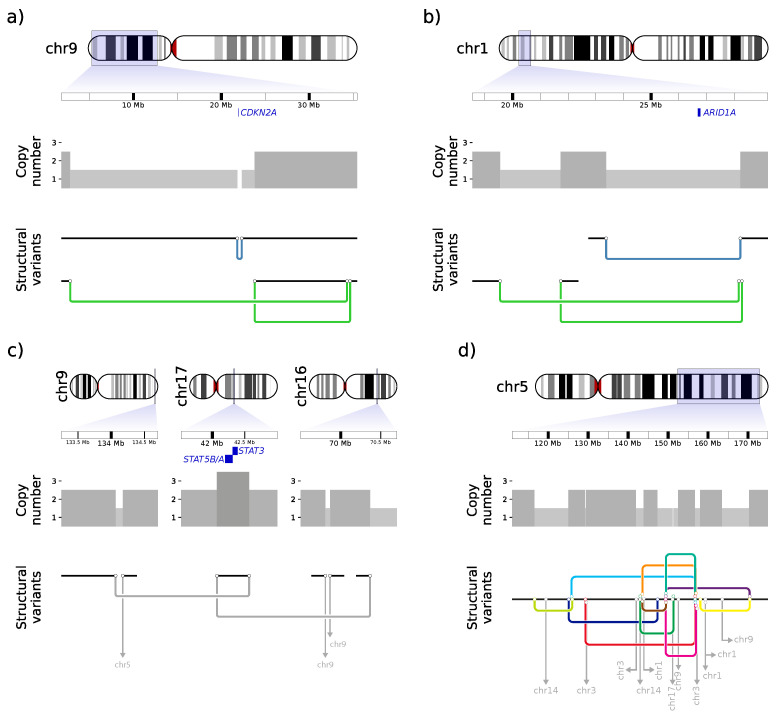
Deletion of *CDKN2A* and *ARID1A*, amplification of *STAT5* by templated insertion SVs and complex rearrangement of chromosome 5. Each panel consists of three parts (top to bottom): The genome position of the respective section with marked position of the gene of interest (blue), the copy-number, and the detected SVs. SVs consist of two breakpoints (black circles) connected by a junction, colored dependent on the type of SV (deletion: blue, inversion: green, translocation: gray). Translocations where only one breakpoint is visible are shown as gray arrows and the chromosomal fusion partner is annotated. Chromosomal segments joined by SVs are depicted by black lines. (**a**) Homozygous deletion of *CDKN2A* by two SVs *in cis*: one small deletion and one bridge of templated insertion SV, where chr9: 23.8–34.4 Mb is inserted inversely in a chr9: 2.8–34.5 Mb deletion. (**b**) Heterozygous deletion of *ARID1A* and an adjacent bridge of templated insertion SV with an insertion of a 2.8 kb segment only 639 bp distant to one *ARID1A* deletion breakpoint. For this SV, single reads spanning both breakpoints as well as the complete inserted region are found. (**c**) Amplification of *STAT3/5* by a chain of templated insertion SV between chr9, chr17, and chr16. (**d**) Complex rearrangement on chr5 with multiple intra- and inter-chromosomal translocations. For a better overview, the SVs are depicted in different colors and SVs with templated insertions are marked by additional breakpoint symbols. Due to the complexity of the region, reconstruction into a potential model was not attempted.

Special attention was directed to the homozygous deletion of the tumor suppressor gene *CDKN2A* already detected in the WXS data (Figure 2b). Genome sequencing revealed three large SVs: one 512 kb deletion and two inversions (10.7 Mb and 31.6 Mb) (Figure 4a). All SVs were validated by Sanger sequencing (Figure S8). The deletion fits to the homozygously deleted region affecting *CDKN2A*. The two inversions are unbalanced, meaning that only one side of the original DNA break is rescued (Figure S9). Both inversions are *in cis* as shown by phasing of targeted long-read sequencing data (Figure S10). This leads to a complex SV consisting of a deletion from 2.8 to 34.5 Mb, visible in the coverage data (Figure 2b), coupled with an inversed insertion of chr9:23.8–34.4 Mb.

Another important CNV found in the WXS data was an amplification on chr17 containing the *STAT3/5* genes. Translocations to chr16 and chr9 are found at the left and right border of the amplified segment, respectively (Figure 4c). If both SVs were *in cis*, the *STAT3/5* amplification would be explained by an SV of the chain of templated insertion class [84]. This would lead to a fusion of chr9 to chr16 with the 495 kb fragment of chr17, containing the CDS for *STAT3/5* in-between. However, *in cis* localization cannot be proven since a long stretch of homozygosity prevents successful phasing here (Figure S10).

A further look into the detected SVs revealed more validated or assumed SVs of the templated insertion class. One validated example of another bridge of templated insertion, with single reads spanning the complete insertion and both adjacent breakpoints, is a 2.2 Mb deletion on chr1 in which a 2.8 kb fragment originally located at chr1:28.2 Mb is inserted (Figure S11). The location of the inserted fragment is extremely close (639 bp) to a breakpoint of another 4.8 Mb deletion, which leads to heterozygous deletion of *ARID1A* (Figure 4b).

In addition to these SVs of the templated insertion class (one assumed and one validated), seven more templated insertion translocations were spanned completely by individual reads (Figure S12). The insertion length was between 219 bp and 4,036 bp. Six of these SVs carried one insertion and one SV had two insertions.

Besides these complicated but (putatively) resolvable SVs, chr5q shows a much more complex rearrangement (Figure 4d). In a 53 Mb region, 15 intrachromosomal and 11 inter-chromosomal translocations to chromosomes 1, 3, 9, and 17 were identified. The high density of breakpoints leading to the same and other chromosomes indicates a chromoplexy-like event for chr5.

Our analysis resolved the causative SVs for some of the most recurrent CNVs in MF, such as the *STAT3/5* gain and *ARID1A* and *CDKN2A* loss. Furthermore, it is exemplary for reconstructing the genomic structure in regions with multiple SVs by combining information on called SVs, copy-number, and (where possible) phasing. We believe this approach to be very important for the CNV-rich disease MF: accurate determination of individual SVs and allelic resolution of SVs (and SNVs) will provide the basis for a deeper understanding of the genomic changes during MF development.

Another important aspect shown in this work is the direct detection on templated insertion SVs in this sample. This class features insertion of one or more genomic segments into a simple translocation, thus leading to its amplification. For the insertion size of this SV, different data are given, ranging from a few bp [85] to 50–100 bp [86], and several Mb [84]. Using short-read sequencing, long templated insertions can only be detected indirectly (e.g., by comparison of allele frequencies [84]). Long-read sequencing has the potential to directly span the complete insertion with one read and validate the templated insertion this way. Thereby, longer reads increase the size of directly detectable templated insertion SVs. Alternatively, phasing of the complete insertion segment or both SV breakpoints into one haplotype enables confident determination of a templated insertion SV. Templated insertion SVs are an erroneous repair product of double-strand breaks [85,86]. As potential mechanisms, the polymerase theta-mediated end-joining pathway [85] or insertion of RNA-derived sequences [86] are discussed. If other MF patients also show multiple templated insertion SVs, this SV class and its associated mechanism could be part of the processes leading to the extensive SVs in MF.

Besides the determination of templated insertions, further detection of common mechanisms for SV formation was unsuccessful. In other lymphoid malignancies, such as chronic myeloid leukemia [87] or T-cell acute lymphatic leukemia [88], aberrant recombination-activating gene (RAG) activity and associated breakpoints close to cryptic RAG recognition sites are observed. This enrichment was discussed in CTCL [20], but no significant enrichment of cryptic RAG recognition sites are detected in this sample (Figure S13). While some SVs show signs of microhomology [89,90] (Figure S14) as a whole, no significant enrichment of microhomology is seen compared to a random background (Figure S13). The question of the mechanism behind SV formation in MF remains open, as does the question of whether it is one predominant mechanism or a mixture of different pathways. To tackle this question, a larger cohort of WGS with accurately called SVs seems necessary. Further information, e.g., regarding the 3D structure [91] or the temporal sequence of individual mutation processes [68], might have to be integrated to obtain a complete picture. In addition to the large SVs, we identified TCR rearrangements for the gamma chain. We assembled the two predominant alleles of this sample (TRGV10-TRGJ1 and TRGV8-TRGJP1) (Figure S15). Unfortunately, the reads from this library are too short for both phasing and assembly of the complete TCR gamma region or the more complex TCR beta region (data not shown). Using native long reads, such an assembly is possible and revealed large SVs, including insertions of additional V-segments [92]. As such, a more extensive analysis of germline structure of MF’s patients TCRs would be feasible and of interest.

## 4. Conclusions

Genomic data for MF account for only a subproportional share of the complete knowledge of genomic changes in CTCL, in particular for early-stage MF. One major obstacle in elucidating the genetic changes in this underrepresented part of CTCL is the low tumor fraction in early-stage MF, which makes the detection of somatic variants and especially CNVs difficult. In this work, we outlined a workflow consisting of dissociation of the skin biopsy and subsequent antigen-based cell capture, thereby increasing tumor fraction and enabling high-sensitivity CNV calling. Using this workflow with CD3+ based enrichment for an early-stage MF sample, the tumor fraction more than doubled. This improvement allowed high-quality calling of the extremely complex CNV landscape of this sample, which was not visible without enrichment. Specific examples only detectable in the enriched sample are the homozygous deletion of *CDKN2A* and focal amplification of *STAT3/5*. The sample preparation presented in this study makes MF samples from early stages more accessible for genetic diagnostics. In future work, this enables the exploration of genetic differences, including the highly frequent and recurrent CNVs, between indolent and progressive patients already in early stages of MF. In addition, we applied long-read nanopore sequencing as a tool for detection of complex SVs. Our results elucidated the SVs leading to recurrent CNVs in MF, namely *ARID1A* deletion and *STAT3/5* amplification. Furthermore, long reads allow phasing of multiple SVs into complex haplotypes. Thereby, we succeeded in resolving the deletion of *CDKN2A* as well as identifying multiple templated insertion SVs. Our work introduces an efficacious methodology for unraveling the genetic changes in low tumor fraction MF samples, either at CNV level by WXS or by complete resolution of SVs using long-read WGS. Due to the simplicity of the technical set-up and the importance of CNVs and SVs in MF, our method is directly applicable in a large cohort study of early-stage MF patients to expand knowledge on the genetic landscape of MF.

## Data Availability

For tumor cell fraction in MF and SS, the dbGaP datasets phs000913, phs000725 and the SRA datasets SRP058948 and SRP059214 were used with appropriate permissions. The data generated during this study are available on request from the corresponding author.

## Supplementary Materials

The following supporting information can be downloaded at: https://www.mdpi.com/article/10.3390/cancers14184466/s1, Figure S1: Histology of the MF skin lesion and picture of the patient’s skin; Figure S2: Genomic copy-umber landscape of the early-stage MF sample; Figure S3: Correlation between the copy-ratios of the CD3+ and the CD4+ samples; Figure S4: CNV calls of chr9p by different CNV callers; Figure S5: Copy-number variation data obtained from whole-exome sequencing from the unprocessed (top in each panel) and CD3+ (bottom in each panel) sample of early-stage MF from different genomic locations; Figure S6: Copy-ratio distribution of individual copy-number states and depiction of signal and noise for copy-ratio data; Figure S7: Size distribution of germline SVs in dbVar Common Structural Variants; Figure S8: Sanger sequencing chromatograms for several detected SVs; Figure S9: Reads supporting the structural variants leading to a homozygous *CDKN2A* deletion; Figure S10: Read phasing can show *in cis* localization of multiple SVs; Figure S11: Supporting evidence for the 2.2 Mb deletion with additional 2.8 kb insertion on chr1; Figure S12: Templated insertion SV with supporting reads; Figure S13: No enrichment of RAG Heptamers (a) or microhomology (b) at SV breakpoints; Figure S14: Putative 8 bp microhomology on the flanks of a deletion on chr20 (a) and best match for homology detection in the *CDKN2A* affecting deletion on chr9 (b); Figure S15: Rearrangement of T-cell receptor gamma. Table S1: Primers used for amplification of selected breakpoints and subsequent Sanger sequencing. Table S2: Regions targeted by adaptive sampling Oxford Nanopore sequencing.

## Abbreviations

The following abbreviations are used in this manuscript:

CTCL	Cutaneous T-cell lymphoma
MF	Mycosis fungoides
SS	Sézary syndrome
CNV	Copy-number variation
SNV	Single nucleotide variation
SV	Structural variation
Mb	Megabase(s)
kb	Kilobase(s)
bp	Base pair(s)
WXS	Whole-exome sequencing
WGS	Whole-genome sequencing
TCR	T-cell receptor

## References

[1] Girardi M., Heald P.W., Wilson L.D. (2004). The pathogenesis of mycosis fungoides. N. Engl. J. Med..

[2] Dummer R., Vermeer M.H., Scarisbrick J.J., Kim Y.H., Stonesifer C., Tensen C.P., Geskin L.J., Quaglino P., Ramelyte E. (2021). Cutaneous T cell lymphoma. Nat. Rev. Dis. Prim..

[3] Kempf W., Mitteldorf C. (2021). Cutaneous T-cell lymphomas-An update 2021. Hematol. Oncol..

[4] Krejsgaard T., Lindahl L.M., Mongan N.P., Wasik M.A., Litvinov I.V., Iversen L., Langhoff E., Woetmann A., Odum N. (2017). Malignant inflammation in cutaneous T-cell lymphoma-a hostile takeover. Semin. Immunopathol..

[5] Beyer M., Möbs M., Humme D., Sterry W. (2011). Pathogenesis of Mycosis fungoides. J. Ger. Soc. Dermatol..

[6] Hodak E., Sherman S., Papadavid E., Bagot M., Querfeld C., Quaglino P., Prince H.M., Ortiz-Romero P.L., Stadler R., Knobler R. (2021). Should we be imaging lymph nodes at initial diagnosis of early-stage mycosis fungoides? Results from the PROspective Cutaneous Lymphoma International Prognostic Index (PROCLIPI) international study. Br. J. Dermatol..

[7] Agar N.S., Wedgeworth E., Crichton S., Mitchell T.J., Cox M., Ferreira S., Robson A., Calonje E., Stefanato C.M., Wain E.M. (2010). Survival outcomes and prognostic factors in mycosis fungoides/Sézary syndrome: Validation of the revised International Society for Cutaneous Lymphomas/European Organisation for Research and Treatment of Cancer staging proposal. J. Clin. Oncol. Off. J. Am. Soc. Clin. Oncol..

[8] de Masson A., O’Malley J.T., Elco C.P., Garcia S.S., Divito S.J., Lowry E.L., Tawa M., Fisher D.C., Devlin P.M., Teague J.E. (2018). High-throughput sequencing of the T cell receptor *β* gene identifies aggressive early-stage mycosis fungoides. Sci. Transl. Med..

[9] Benton E.C., Crichton S., Talpur R., Agar N.S., Fields P.A., Wedgeworth E., Mitchell T.J., Cox M., Ferreira S., Liu P. (2013). A cutaneous lymphoma international prognostic index (CLIPi) for mycosis fungoides and Sezary syndrome. Eur. J. Cancer.

[10] Scarisbrick J.J., Quaglino P., Prince H.M., Papadavid E., Hodak E., Bagot M., Servitje O., Berti E., Ortiz-Romero P., Stadler R. (2019). The PROCLIPI international registry of early-stage mycosis fungoides identifies substantial diagnostic delay in most patients. Br. J. Dermatol..

[11] Dippel E., Assaf C., Becker J.C., von Bergwelt-Baildon M., Bernreiter S., Cozzio A., Eich H.T., Elsayad K., Follmann M., Grabbe S. (2022). S2k-Leitlinie - Kutane Lymphome (ICD10 C82-C86): Update 2021. J. Ger. Soc. Dermatol..

[12] Wilcox R.A. (2017). Cutaneous T-cell lymphoma: 2017 update on diagnosis, risk-stratification, and management. Am. J. Hematol..

[13] Stadler R., Scarisbrick J.J. (2021). Maintenance therapy in patients with mycosis fungoides or Sézary syndrome: A neglected topic. Eur. J. Cancer.

[14] Horwitz S.M., Scarisbrick J.J., Dummer R., Whittaker S., Duvic M., Kim Y.H., Quaglino P., Zinzani P.L., Bechter O., Eradat H. (2021). Randomized phase 3 ALCANZA study of brentuximab vedotin vs physician’s choice in cutaneous T-cell lymphoma: Final data. Blood Adv..

[15] Kim Y.H., Bagot M., Pinter-Brown L., Rook A.H., Porcu P., Horwitz S.M., Whittaker S., Tokura Y., Vermeer M., Zinzani P.L. (2018). Mogamulizumab versus vorinostat in previously treated cutaneous T-cell lymphoma (MAVORIC): An international, open-label, randomised, controlled phase 3 trial. Lancet Oncol..

[16] DiNardo C.D., Cortes J.E. (2016). Mutations in AML: Prognostic and therapeutic implications. Hematol. Am. Soc. Hematol. Educ. Program.

[17] Lopez-Santillan M., Lopez-Lopez E., Alvarez-Gonzalez P., Martinez G., Arzuaga-Mendez J., Ruiz-Diaz I., Guerra-Merino I., Gutierrez-Camino A., Martin-Guerrero I. (2021). Prognostic and therapeutic value of somatic mutations in diffuse large B-cell lymphoma: A systematic review. Crit. Rev. Oncol..

[18] Griffith O.L., Spies N.C., Anurag M., Griffith M., Luo J., Tu D., Yeo B., Kunisaki J., Miller C.A., Krysiak K. (2018). The prognostic effects of somatic mutations in ER-positive breast cancer. Nat. Commun..

[19] Bastidas Torres A.N., Cats D., Mei H., Szuhai K., Willemze R., Vermeer M.H., Tensen C.P. (2018). Genomic analysis reveals recurrent deletion of JAK-STAT signaling inhibitors HNRNPK and SOCS1 in mycosis fungoides. Genes Chromosom. Cancer.

[20] Choi J., Goh G., Walradt T., Hong B.S., Bunick C.G., Chen K., Bjornson R.D., Maman Y., Wang T., Tordoff J. (2015). Genomic landscape of cutaneous T cell lymphoma. Nat. Genet..

[21] da Silva Almeida A.C., Abate F., Khiabanian H., Martinez-Escala E., Guitart J., Tensen C.P., Vermeer M.H., Rabadan R., Ferrando A., Palomero T. (2015). The mutational landscape of cutaneous T cell lymphoma and Sézary syndrome. Nat. Genet..

[22] McGirt L.Y., Jia P., Baerenwald D.A., Duszynski R.J., Dahlman K.B., Zic J.A., Zwerner J.P., Hucks D., Dave U., Zhao Z. (2015). Whole-genome sequencing reveals oncogenic mutations in mycosis fungoides. Blood.

[23] Ungewickell A., Bhaduri A., Rios E., Reuter J., Lee C.S., Mah A., Zehnder A., Ohgami R., Kulkarni S., Armstrong R. (2015). Genomic analysis of mycosis fungoides and Sézary syndrome identifies recurrent alterations in TNFR2. Nat. Genet..

[24] Kiel M.J., Sahasrabuddhe A.A., Rolland D.C.M., Velusamy T., Chung F., Schaller M., Bailey N.G., Betz B.L., Miranda R.N., Porcu P. (2015). Genomic analyses reveal recurrent mutations in epigenetic modifiers and the JAK-STAT pathway in Sézary syndrome. Nat. Commun..

[25] Wang L., Ni X., Covington K.R., Yang B.Y., Shiu J., Zhang X., Xi L., Meng Q., Langridge T., Drummond J. (2015). Genomic profiling of Sézary syndrome identifies alterations of key T cell signaling and differentiation genes. Nat. Genet..

[26] Woollard W.J., Pullabhatla V., Lorenc A., Patel V.M., Butler R.M., Bayega A., Begum N., Bakr F., Dedhia K., Fisher J. (2016). Candidate driver genes involved in genome maintenance and DNA repair in Sézary syndrome. Blood.

[27] Prasad A., Rabionet R., Espinet B., Zapata L., Puiggros A., Melero C., Puig A., Sarria-Trujillo Y., Ossowski S., Garcia-Muret M.P. (2016). Identification of Gene Mutations and Fusion Genes in Patients with Sézary Syndrome. J. Investig. Dermatol..

[28] Iyer A., Hennessey D., O’Keefe S., Patterson J., Wang W., Wong G.K.S., Gniadecki R. (2020). Independent evolution of cutaneous lymphoma subclones in different microenvironments of the skin. Sci. Rep..

[29] Iżykowska K., Przybylski G.K., Gand C., Braun F.C., Grabarczyk P., Kuss A.W., Olek-Hrab K., Bastidas Torres A.N., Vermeer M.H., Zoutman W.H. (2017). Genetic rearrangements result in altered gene expression and novel fusion transcripts in Sézary syndrome. Oncotarget.

[30] Sekulic A., Liang W.S., Tembe W., Izatt T., Kruglyak S., Kiefer J.A., Cuyugan L., Zismann V., Legendre C., Pittelkow M.R. (2015). Personalized treatment of Sézary syndrome by targeting a novel CTLA4:CD28 fusion. Mol. Genet. Genom. Med..

[31] Benavides-Huerto M.A., Paredes-Solís V., Lagunas-Rangel F.A. (2019). Mycosis fungoides: A challenge for the diagnosis. J. Oncol. Sci..

[32] Stolearenco V., Namini M.R.J., Hasselager S.S., Gluud M., Buus T.B., Willerslev-Olsen A., dum N., Krejsgaard T. (2020). Cellular Interactions and Inflammation in the Pathogenesis of Cutaneous T-Cell Lymphoma. Front. Cell Dev. Biol..

[33] Humme D., Lukowsky A., Gierisch M., Haider A., Vandersee S., Assaf C., Sterry W., Möbs M., Beyer M. (2014). T-cell receptor gene rearrangement analysis of sequential biopsies in cutaneous T-cell lymphomas with the Biomed-2 PCR reveals transient T-cell clones in addition to the tumor clone. Exp. Dermatol..

[34] Yamashita T., Abbade L.P.F., Marques M.E.A., Marques S.A. (2012). Mycosis fungoides and Sézary syndrome: Clinical, histopathological and immunohistochemical review and update. An. Bras. Dermatol..

[35] Iyer A., Hennessey D., O’Keefe S., Patterson J., Wang W., Wong G.K.S., Gniadecki R. (2020). Branched evolution and genomic intratumor heterogeneity in the pathogenesis of cutaneous T-cell lymphoma. Blood Adv..

[36] Miura T., Yasuda S., Sato Y. (2021). A simple method to estimate the in-house limit of detection for genetic mutations with low allele frequencies in whole-exome sequencing analysis by next-generation sequencing. BMC Genom. Data.

[37] Lier A., Penzel R., Heining C., Horak P., Fröhlich M., Uhrig S., Budczies J., Kirchner M., Volckmar A.L., Hutter B. (2018). Validating Comprehensive Next-Generation Sequencing Results for Precision Oncology: The NCT/DKTK Molecularly Aided Stratification for Tumor Eradication Research Experience. JCO Precis. Oncol..

[38] Yan Y.H., Chen S.X., Cheng L.Y., Rodriguez A.Y., Tang R., Cabrera K., Zhang D.Y. (2021). Confirming putative variants at ≤5% allele frequency using allele enrichment and Sanger sequencing. Sci. Rep..

[39] Cortés-Ciriano I., Gulhan D.C., Lee J.J.K., Melloni G.E.M., Park P.J. (2022). Computational analysis of cancer genome sequencing data. Nat. Rev. Genet..

[40] Gabrielaite M., Torp M.H., Rasmussen M.S., Andreu-Sánchez S., Vieira F.G., Pedersen C.B., Kinalis S., Madsen M.B., Kodama M., Demircan G.S. (2021). A Comparison of Tools for Copy-Number Variation Detection in Germline Whole Exome and Whole Genome Sequencing Data. Cancers.

[41] Gordeeva V., Sharova E., Babalyan K., Sultanov R., Govorun V.M., Arapidi G. (2021). Benchmarking germline CNV calling tools from exome sequencing data. Sci. Rep..

[42] Zhao L., Liu H., Yuan X., Gao K., Duan J. (2020). Comparative study of whole exome sequencing-based copy number variation detection tools. BMC Bioinform..

[43] van Belzen I.A.E.M., Schönhuth A., Kemmeren P., Hehir-Kwa J.Y. (2021). Structural variant detection in cancer genomes: Computational challenges and perspectives for precision oncology. NPJ Precis. Oncol..

[44] Roberts H.E., Lopopolo M., Pagnamenta A.T., Sharma E., Parkes D., Lonie L., Freeman C., Knight S.J.L., Lunter G., Dreau H. (2021). Short and long-read genome sequencing methodologies for somatic variant detection; genomic analysis of a patient with diffuse large B-cell lymphoma. Sci. Rep..

[45] Mahmoud M., Gobet N., Cruz-Dávalos D.I., Mounier N., Dessimoz C., Sedlazeck F.J. (2019). Structural variant calling: The long and the short of it. Genome Biol..

[46] Cretu Stancu M., van Roosmalen M.J., Renkens I., Nieboer M.M., Middelkamp S., de Ligt J., Pregno G., Giachino D., Mandrile G., Espejo Valle-Inclan J. (2017). Mapping and phasing of structural variation in patient genomes using nanopore sequencing. Nat. Commun..

[47] Gong L., Wong C.H., Cheng W.C., Tjong H., Menghi F., Ngan C.Y., Liu E.T., Wei C.L. (2018). Picky comprehensively detects high-resolution structural variants in nanopore long reads. Nat. Methods.

[48] Miller D.E., Sulovari A., Wang T., Loucks H., Hoekzema K., Munson K.M., Lewis A.P., Fuerte E.P.A., Paschal C.R., Thies J. (2020). Targeted Long-Read Sequencing Resolves Complex Structural Variants and Identifies Missing Disease-Causing Variants.

[49] Wang Y., Zhao Y., Bollas A., Wang Y., Au K.F. (2021). Nanopore sequencing technology, bioinformatics and applications. Nat. Biotechnol..

[50] Hain C., Stadler R., Kalinowski J. (2021). Sézary Syndrome Shows Whole Genome Duplication as a Late Event in Tumor Evolution. J. Investig. Dermatol..

[51] van der Auwera G., O’Connor B.D. (2020). Genomics in the Cloud: Using Docker, GATK, and WDL in Terra.

[52] Talevich E., Shain A.H., Botton T., Bastian B.C. (2016). CNVkit: Genome-Wide Copy Number Detection and Visualization from Targeted DNA Sequencing. PLoS Comput. Biol..

[53] Li J., Lupat R., Amarasinghe K.C., Thompson E.R., Doyle M.A., Ryland G.L., Tothill R.W., Halgamuge S.K., Campbell I.G., Gorringe K.L. (2012). CONTRA: Copy number analysis for targeted resequencing. Bioinformatics.

[54] Wick R.R., Judd L.M., Gorrie C.L., Holt K.E. (2017). Completing bacterial genome assemblies with multiplex MinION sequencing. Microb. Genom..

[55] Li H. (2018). Minimap2: Pairwise alignment for nucleotide sequences. Bioinformatics.

[56] Sedlazeck F.J., Rescheneder P., Smolka M., Fang H., Nattestad M., von Haeseler A., Schatz M.C. (2018). Accurate detection of complex structural variations using single-molecule sequencing. Nat. Methods.

[57] Smolka M., Paulin L.F., Grochowski C.M., Mahmoud M., Behera S., Gandhi M., Hong K., Pehlivan D., Scholz S.W., Carvalho C.M. (2022). Comprehensive Structural Variant Detection: From Mosaic to Population-Level.

[58] Krzywinski M., Schein J., Birol I., Connors J., Gascoyne R., Horsman D., Jones S.J., Marra M.A. (2009). Circos: An information aesthetic for comparative genomics. Genome Res..

[59] Edge P., Bansal V. (2019). Longshot enables accurate variant calling in diploid genomes from single-molecule long read sequencing. Nat. Commun..

[60] Martin M., Patterson M., Garg S., O Fischer S., Pisanti N., Klau G.W., Schöenhuth A., Marschall T. (2016). WhatsHap: Fast and Accurate Read-Based Phasing. BioRxiv.

[61] Frith M.C., Mitsuhashi S., Katoh K. (2021). lamassemble: Multiple Alignment and Consensus Sequence of Long Reads. Methods Mol. Biol..

[62] Bolotin D.A., Poslavsky S., Mitrophanov I., Shugay M., Mamedov I.Z., Putintseva E.V., Chudakov D.M. (2015). MiXCR: Software for comprehensive adaptive immunity profiling. Nat. Methods.

[63] Carter S.L., Cibulskis K., Helman E., McKenna A., Shen H., Zack T., Laird P.W., Onofrio R.C., Winckler W., Weir B.A. (2012). Absolute quantification of somatic DNA alterations in human cancer. Nat. Biotechnol..

[64] Hristov A.C., Tejasvi T., Wilcox R.A. (2019). Mycosis fungoides and Sézary syndrome: 2019 update on diagnosis, risk-stratification, and management. Am. J. Hematol..

[65] Florell S.R., Cessna M., Lundell R.B., Boucher K.M., Bowen G.M., Harris R.M., Petersen M.J., Zone J.J., Tripp S., Perkins S.L. (2006). Usefulness (or Lack Thereof) of Immunophenotyping in Atypical Cutaneous T-Cell Infiltrates. Am. J. Clin. Pathol..

[66] Tirumalae R., Panjwani P.K. (2012). Origin Use of CD4, CD8, and CD1a Immunostains in Distinguishing Mycosis Fungoides from its Inflammatory Mimics: A Pilot Study. Indian J. Dermatol..

[67] Hodak E., David M., Maron L., Aviram A., Kaganovsky E., Feinmesser M. (2006). CD4/CD8 double-negative epidermotropic cutaneous T-cell lymphoma: An immunohistochemical variant of mycosis fungoides. J. Am. Acad. Dermatol..

[68] Gerstung M., Jolly C., Leshchiner I., Dentro S.C., Gonzalez S., Rosebrock D., Mitchell T.J., Rubanova Y., Anur P., Yu K. (2020). The evolutionary history of 2,658 cancers. Nature.

[69] Nagata Y., Kontani K., Enami T., Kataoka K., Ishii R., Totoki Y., Kataoka T.R., Hirata M., Aoki K., Nakano K. (2016). Variegated RHOA mutations in adult T-cell leukemia/lymphoma. Blood.

[70] Park J., Yang J., Wenzel A.T., Ramachandran A., Lee W.J., Daniels J.C., Kim J., Martinez-Escala E., Amankulor N., Pro B. (2017). Genomic analysis of 220 CTCLs identifies a novel recurrent gain-of-function alteration in RLTPR (p.Q575E). Blood.

[71] Brenan L., Andreev A., Cohen O., Pantel S., Kamburov A., Cacchiarelli D., Persky N.S., Zhu C., Bagul M., Goetz E.M. (2016). Phenotypic Characterization of a Comprehensive Set of MAPK1/ERK2 Missense Mutants. Cell Rep..

[72] Gug G., Huang Q., Chiticariu E., Solovan C., Baudis M. (2019). DNA copy number imbalances in primary cutaneous lymphomas. J. Eur. Acad. Dermatol. Venereol..

[73] Scarisbrick J.J., Woolford A.J., Russell-Jones R., Whittaker S.J. (2001). Allelotyping in mycosis fungoides and Sézary syndrome: Common regions of allelic loss identified on 9p, 10q, and 17p. J. Investig. Dermatol..

[74] Meggendorfer M., Haferlach C., Zenger M., Macijewski K., Kern W., Haferlach T. (2016). The landscape of myeloid neoplasms with isochromosome 17q discloses a specific mutation profile and is characterized by an accumulation of prognostically adverse molecular markers. Leukemia.

[75] Barbouti A., Stankiewicz P., Nusbaum C., Cuomo C., Cook A., Höglund M., Johansson B., Hagemeijer A., Park S.S., Mitelman F. (2004). The breakpoint region of the most common isochromosome, i(17q), in human neoplasia is characterized by a complex genomic architecture with large, palindromic, low-copy repeats. Am. J. Hum. Genet..

[76] Inoue K., Fry E.A. (2017). Haploinsufficient tumor suppressor genes. Adv. Med. Biol..

[77] Braun M., Pastorczak A., Fendler W., Madzio J., Tomasik B., Taha J., Bielska M., Sedek L., Szczepanski T., Matysiak M. (2017). Biallelic loss of CDKN2A is associated with poor response to treatment in pediatric acute lymphoblastic leukemia. Leuk. Lymphoma.

[78] Iacobucci I., Ferrari A., Lonetti A., Papayannidis C., Paoloni F., Trino S., Storlazzi C.T., Ottaviani E., Cattina F., Impera L. (2011). CDKN2A/B alterations impair prognosis in adult BCR-ABL1-positive acute lymphoblastic leukemia patients. Clin. Cancer Res. Off. J. Am. Assoc. Cancer Res..

[79] Gallucci M., Vico E., Merola R., Leonardo C., Sperduti I., Felici A., Sentinelli S., Cantiani R., Orlandi G., Cianciulli A. (2007). Adverse genetic prognostic profiles define a poor outcome for cystectomy in bladder cancer. Exp. Mol. Pathol..

[80] Stengel A., Schnittger S., Weissmann S., Kuznia S., Kern W., Kohlmann A., Haferlach T., Haferlach C. (2014). TP53 mutations occur in 15.7% of ALL and are associated with MYC-rearrangement, low hypodiploidy, and a poor prognosis. Blood.

[81] Marshall A.E., Roes M.V., Passos D.T., DeWeerd M.C., Chaikovsky A.C., Sage J., Howlett C.J., Dick F.A. (2019). RB1 Deletion in Retinoblastoma Protein Pathway-Disrupted Cells Results in DNA Damage and Cancer Progression. Mol. Cell. Biol..

[82] Sun X., Wang S.C., Wei Y., Luo X., Jia Y., Li L., Gopal P., Zhu M., Nassour I., Chuang J.C. (2017). Arid1a Has Context-Dependent Oncogenic and Tumor Suppressor Functions in Liver Cancer. Cancer Cell.

[83] Liu Y., Rosikiewicz W., Pan Z., Jillette N., Wang P., Taghbalout A., Foox J., Mason C., Carroll M., Cheng A. (2021). DNA methylation-calling tools for Oxford Nanopore sequencing: A survey and human epigenome-wide evaluation. Genome Biol..

[84] Li Y., Roberts N.D., Wala J.A., Shapira O., Schumacher S.E., Kumar K., Khurana E., Waszak S., Korbel J.O., Haber J.E. (2020). Patterns of somatic structural variation in human cancer genomes. Nature.

[85] Schimmel J., van Schendel R., den Dunnen J.T., Tijsterman M. (2019). Templated Insertions: A Smoking Gun for Polymerase Theta-Mediated End Joining. Trends Genet. TIG.

[86] Onozawa M., Zhang Z., Kim Y.J., Goldberg L., Varga T., Bergsagel P.L., Kuehl W.M., Aplan P.D. (2014). Repair of DNA double-strand breaks by templated nucleotide sequence insertions derived from distant regions of the genome. Proc. Natl. Acad. Sci. USA.

[87] Thomson D.W., Shahrin N.H., Wang P.P.S., Wadham C., Shanmuganathan N., Scott H.S., Dinger M.E., Hughes T.P., Schreiber A.W., Branford S. (2020). Aberrant RAG-mediated recombination contributes to multiple structural rearrangements in lymphoid blast crisis of chronic myeloid leukemia. Leukemia.

[88] Raschke S., Balz V., Efferth T., Schulz W.A., Florl A.R. (2005). Homozygous deletions of CDKN2A caused by alternative mechanisms in various human cancer cell lines. Genes Chromosom. Cancer.

[89] Carvajal-Garcia J., Cho J.E., Carvajal-Garcia P., Feng W., Wood R.D., Sekelsky J., Gupta G.P., Roberts S.A., Ramsden D.A. (2020). Mechanistic basis for microhomology identification and genome scarring by polymerase theta. Proc. Natl. Acad. Sci. USA.

[90] Ottaviani D., LeCain M., Sheer D. (2014). The role of microhomology in genomic structural variation. Trends Genet. TIG.

[91] Harewood L., Kishore K., Eldridge M.D., Wingett S., Pearson D., Schoenfelder S., Collins V.P., Fraser P. (2017). Hi-C as a tool for precise detection and characterisation of chromosomal rearrangements and copy number variation in human tumours. Genome Biol..

[92] Zhang J.Y., Roberts H., Flores D.S.C., Cutler A.J., Brown A.C., Whalley J.P., Mielczarek O., Buck D., Lockstone H., Xella B. (2021). Using de novo assembly to identify structural variation of eight complex immune system gene regions. PLoS Comput. Biol..

