# Supplementary material for: Unraveling the Structural Variations of Early-Stage Mycosis Fungoides—CD3 Based Purification and Third Generation Sequencing as Novel Tools for the Genomic Landscape in CTCL
Source: Cancers (Basel). 2022 Sep 14;14(18):4466. doi: 10.3390/cancers14184466 (PMC9497107; doi:10.3390/cancers14184466)
Supplement: Supplementary file 1 [file cancers-14-04466-s001.zip › cancers-1881848-supplementary.pdf]

Unraveling the structural variations of early-stage mycosis fungoides – CD3 based purification and third generation sequencing as novel tools for the genomic landscape in CTCL - Supplementary Material

Carsten Hain, Rudolf Stadler and Jörn Kalinowski

Contents

1 Supplementary Figures **2**

2 Supplementary Tables **15**

1 Supplementary Figures

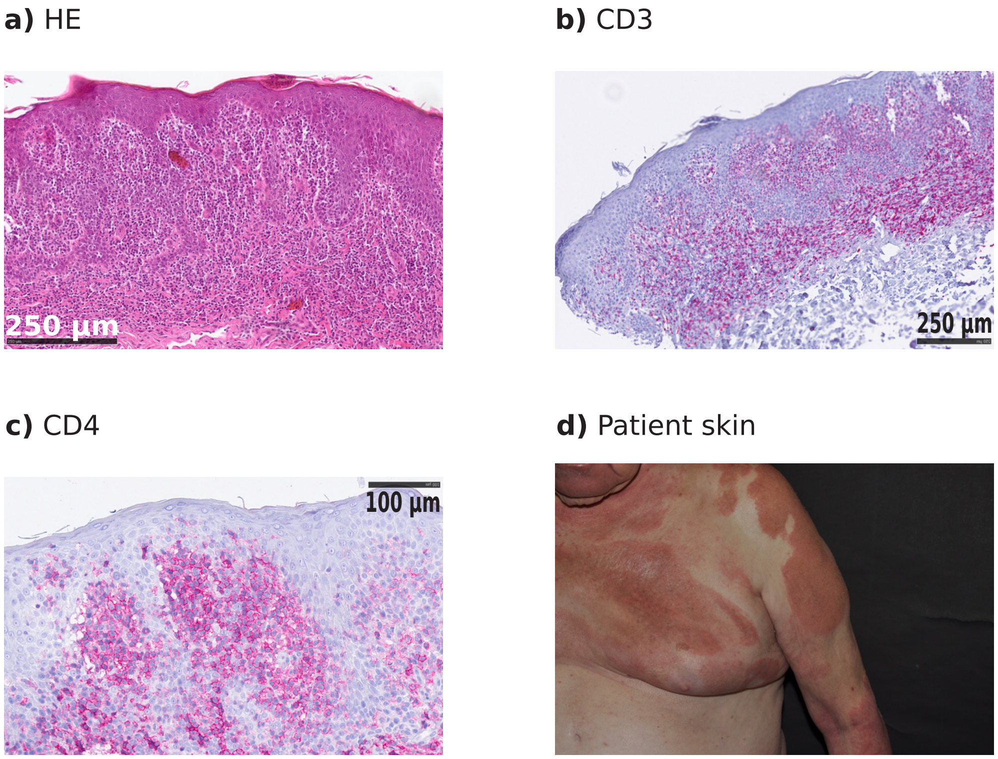

Figure S1: Histology of the MF skin lesion and picture of the patient's skin. Parts of the skin lesion examined in this publication were analyzed by histology. Sections were stained with HE (a) or anti-CD3 (b) or anti-CD4 (c). In the histology pictures, the scale bar is 250 µm in (a) and (b) and 100 µm in (c). Furthermore, (d) gives a representative picture of the patient's skin during the time of the biopsy.

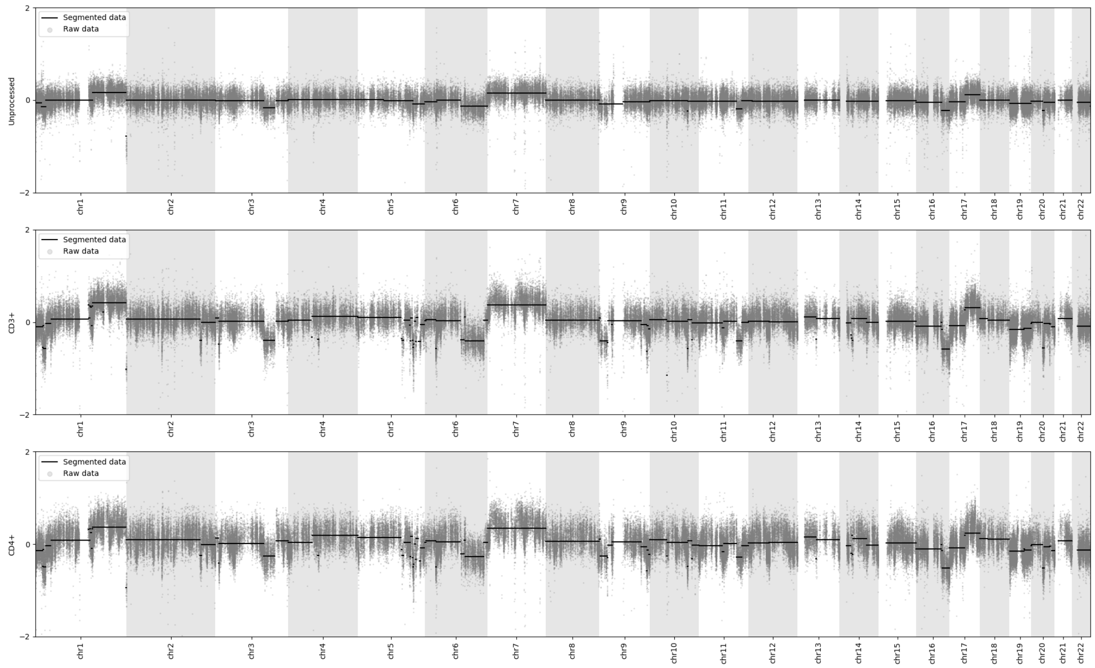

Figure S2: Genomic copy-number landscape of the early-stage MF sample. Copy-number as raw (gray dots) and segmented (black lines) copy-ratio is plotted against the genomic coordinate for the unprocessed sample (top) and the CD3+ (middle) or CD4+ (bottom) enriched cells.

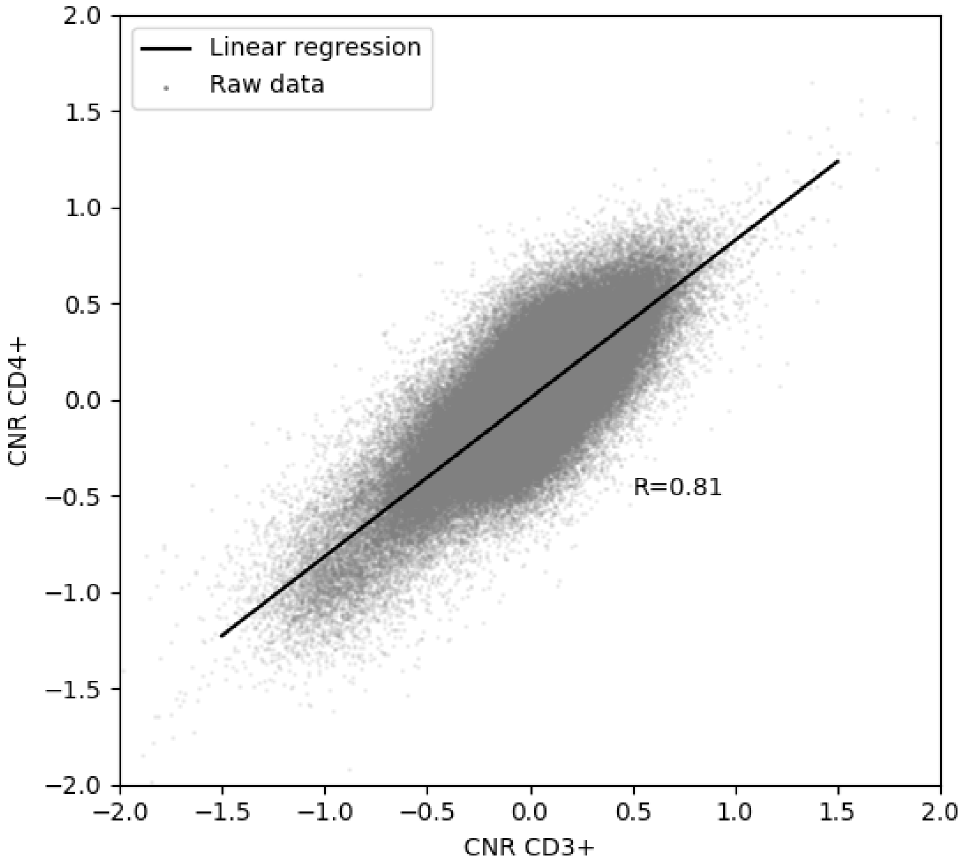

Figure S3: Correlation between the copy-ratios of the CD3+ and the CD4+ samples. Copy-ratio as raw data (gray dots) of the CD3+ and the CD4+ sample were plotted against each other. A linear regression () with Pearons R = 0.81 is shown (black line). Clonal differences between both samples should be visible as clusters off the diagonal.

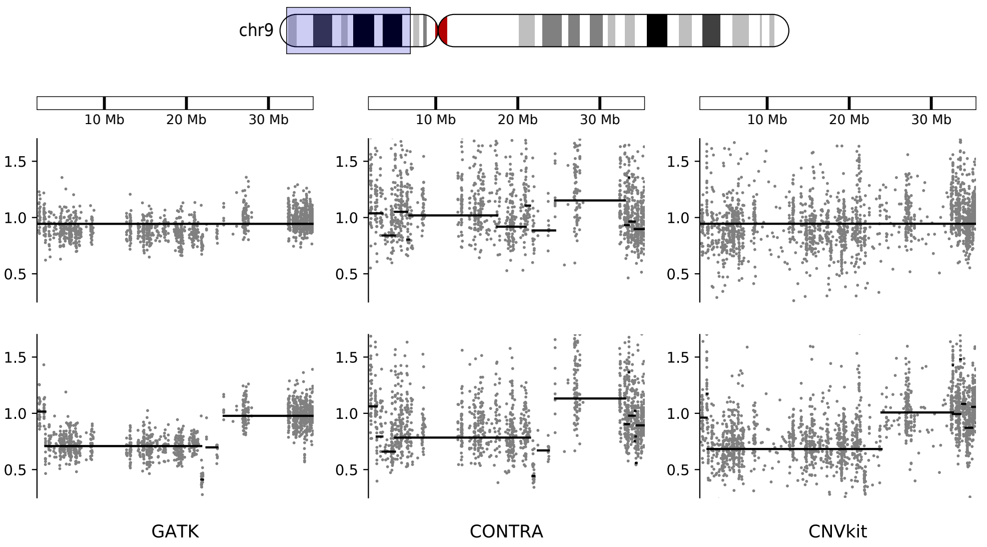

Figure S4: CNV calls of chr9p by different CNV callers. The WXS data from the unprocessed biopsy (top) and the CD3+ cells (bottom) were analyzed for CNVs using the tools GATK, CONTRA and CNVkit. Copy-ratio (gray dots) and called segments (black) lines are shown.

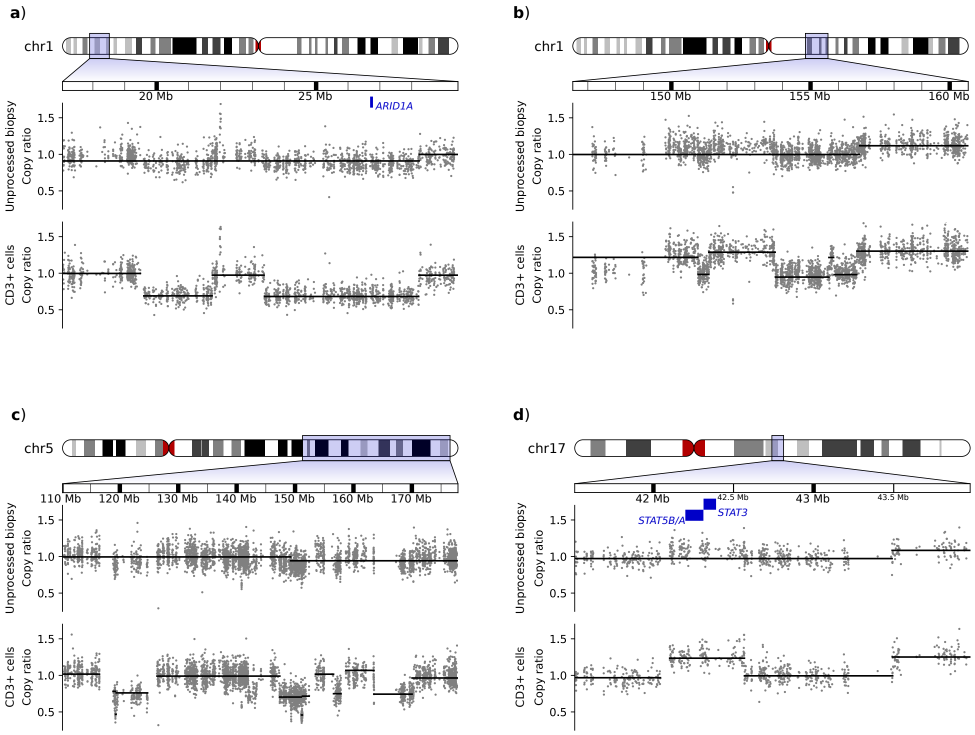

Figure S5: Copy-number variation data obtained from whole-exome sequencing from the unprocessed (top in each panel) and CD3+ (bottom in each panel) sample of early-stage MF from different genomic locations. Half of the tissue-dissociated MF biopsy was used without further processing, while CD3+ cells were enriched in the other half. Both samples were analyzed by whole-exome sequencing and somatic copy-number variation data was calculated. Raw copy-ratio (denoised and normalized read depth of individual exons, gray dots) and modeled CNV calls (black lines) are depicted. (a) Heterozygous deletion of *ARID1A*, multiple CNVs in a small (b) or large (c) genomic region and (d) amplification of *STAT3/5*. The genomic location is indicated above each panel. The genes of interest are showed as colored boxes.

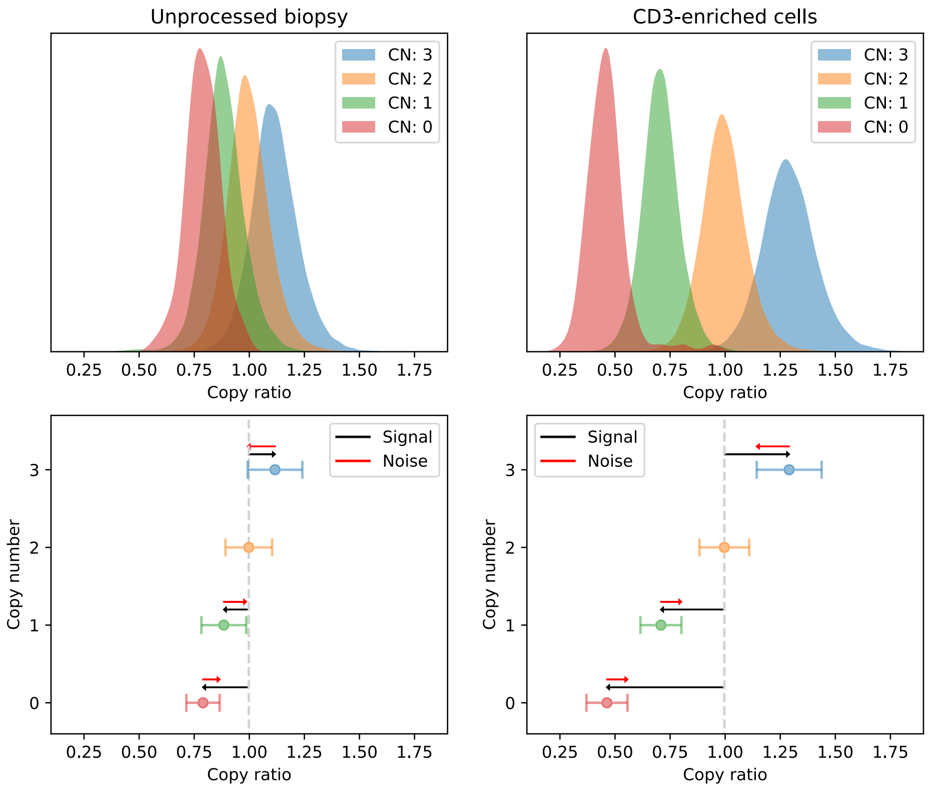

Figure S6: Copy-ratio distribution of individual copy-number states and depiction of signal and noise for copy-ratio data. All single exon copy-ratio data for individual copy-numbers was aggregated and plotted as Gaussian kernel density (top). Copy-number was plotted against copy-ratio means and standard deviations Theoretical CNV calling signal (difference between mean copy-ratio of this copy-number and copy-ratio of copy-number 2) and noise (standard deviation of this copy-number's copy-ratio distribution) are depicted as black and red arrows respectively.

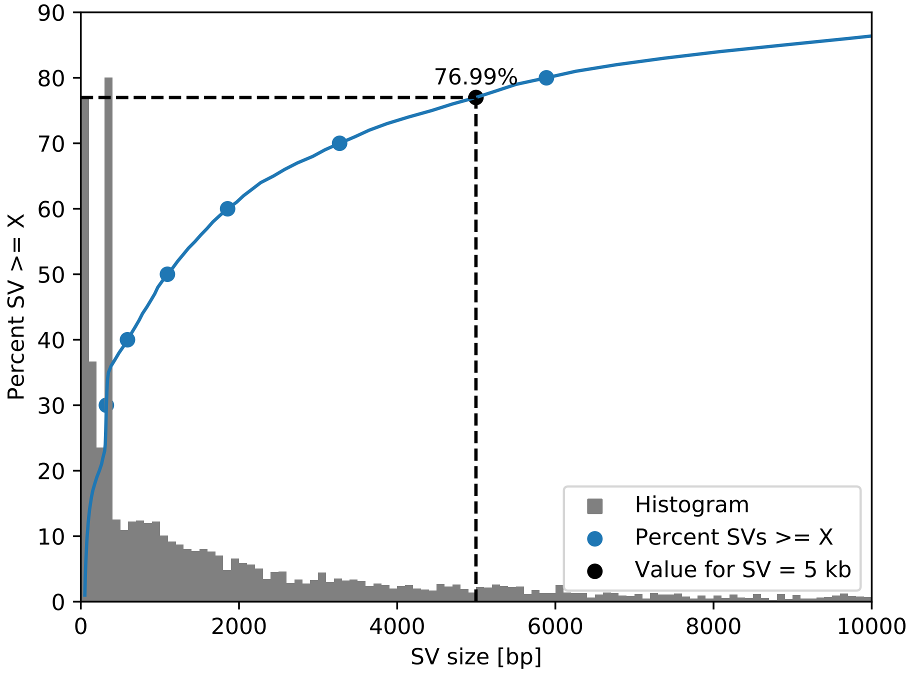

Figure S7: Size distribution of germline SVs in dbVar Common Structural Variants. SV data from the dbVar Common Structural Variants were obtained from UCSC genome browser. SV size is plotted as a histogram (gray) showing that longer SVs are becoming steadily less common. One exception is the peak at around 300 bp indicating deletions/insertions of Alu elements. Cumulative frequency (blue) indicates this trend as well. The chosen cutoff for SV calling of 5 kb removes three quarters of germline SVs (black).

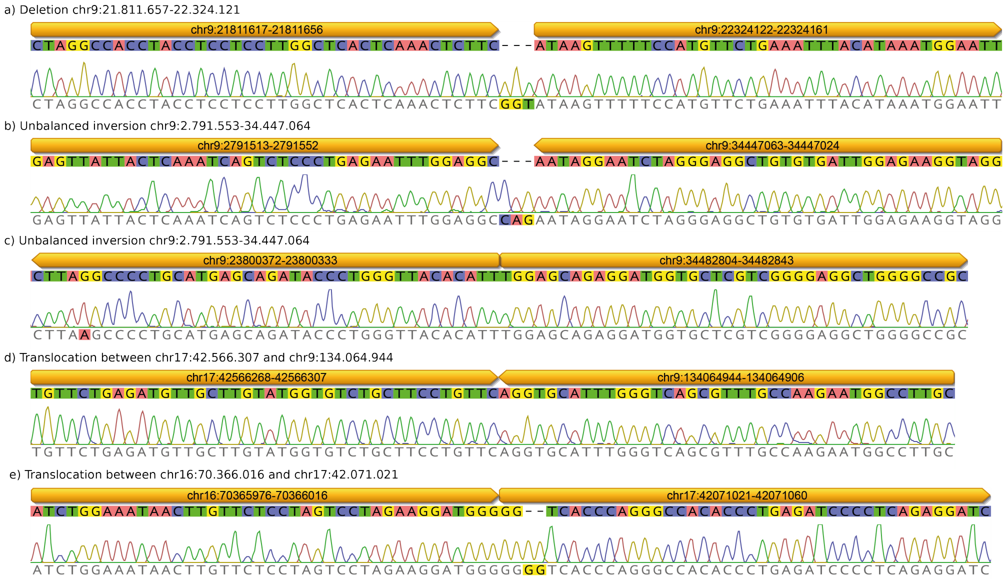

Figure S8: Sanger sequencing chromatograms for several detected SVs.

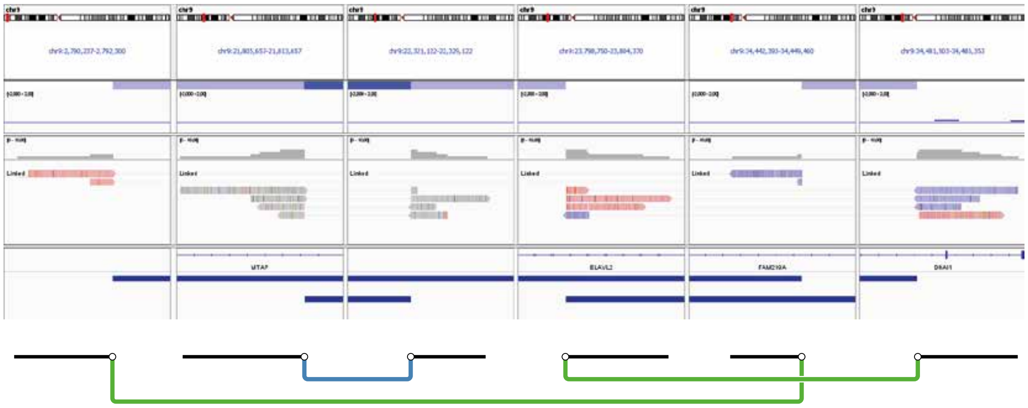

Figure S9: Reads supporting the structural variants leading to a homozygous *CDKN2A* deletion. Reads supporting the three structural variations leading to homozygous loss of *CDKN2A* were mapped to GRCh38. Primary and supplementary mappings are connected by thin lines. The reads, supporting the deletion (panel 2 and 3), are mapping outside on both sides of the deleted region, thus showing the expected pattern for a deletion. The reads supporting the "inversion" (panel 1 and 5 or 4 and 5, respectively) show unexpected behavior as only reads supporting one genomic fusion are present. The orientation of reads with different orientations at different mapping positions are color-coded (red: + orientation; blue: - orientation)

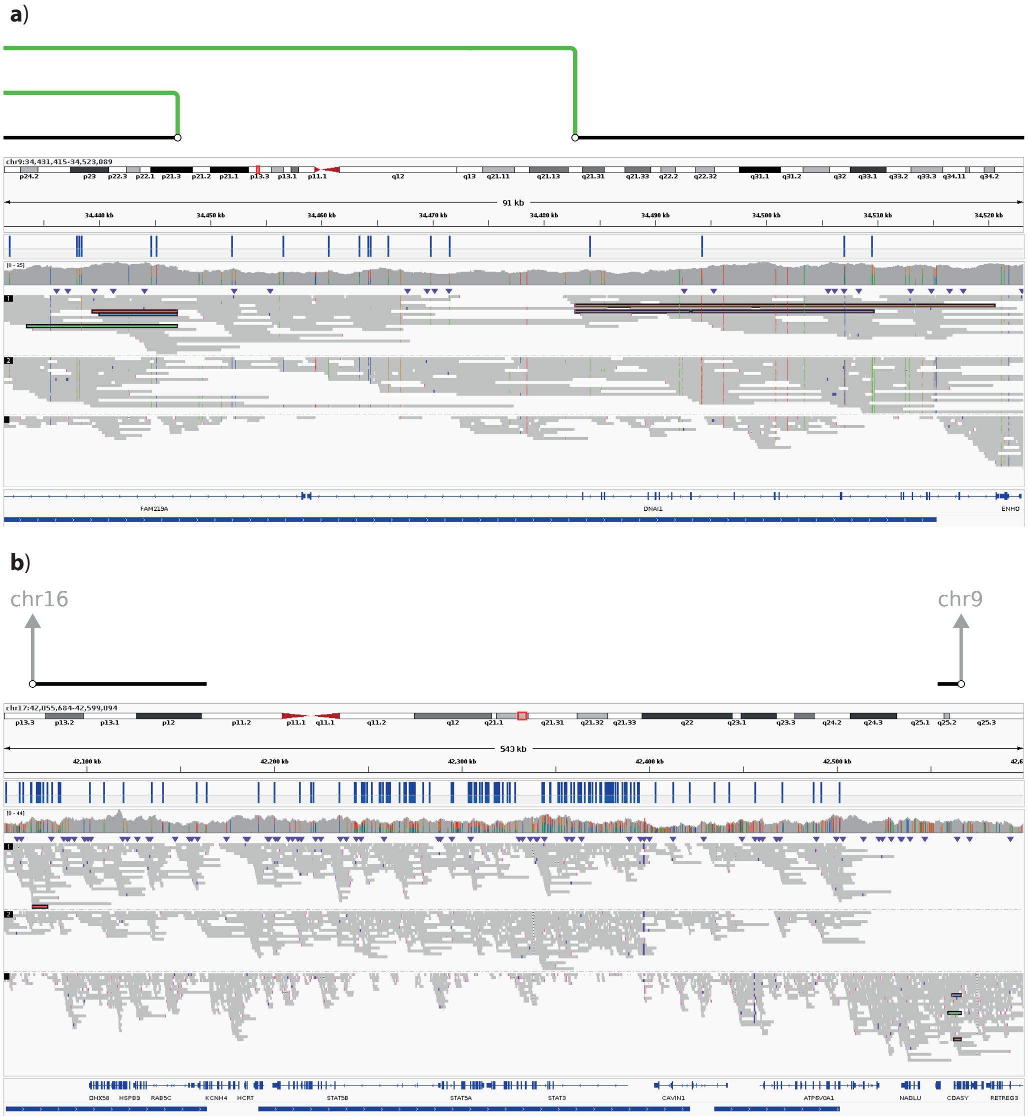

Figure S10: Read phasing can show *in cis* localization of multiple SVs. (a) Verified *in cis* localization of two SVs leading to a long deletion on chr9. (b) Incomplete phasing of both SVs bordering the *STAT3/5* amplification due to a long stretch of homozygosity. Nanopore sequencing data around known SVs were obtained using adaptive sampling from the unprocessed biopsy. The reads were phased using WhatsHap, and reads supporting the indicated SVs are marked. Read are depicted as haplotagged into the phases 1, 2, or undefined. Resulting haploblocks (large blue boxes, bottom) and heterozygous SNPs (small blue boxes, top) are shown.

a)

b)

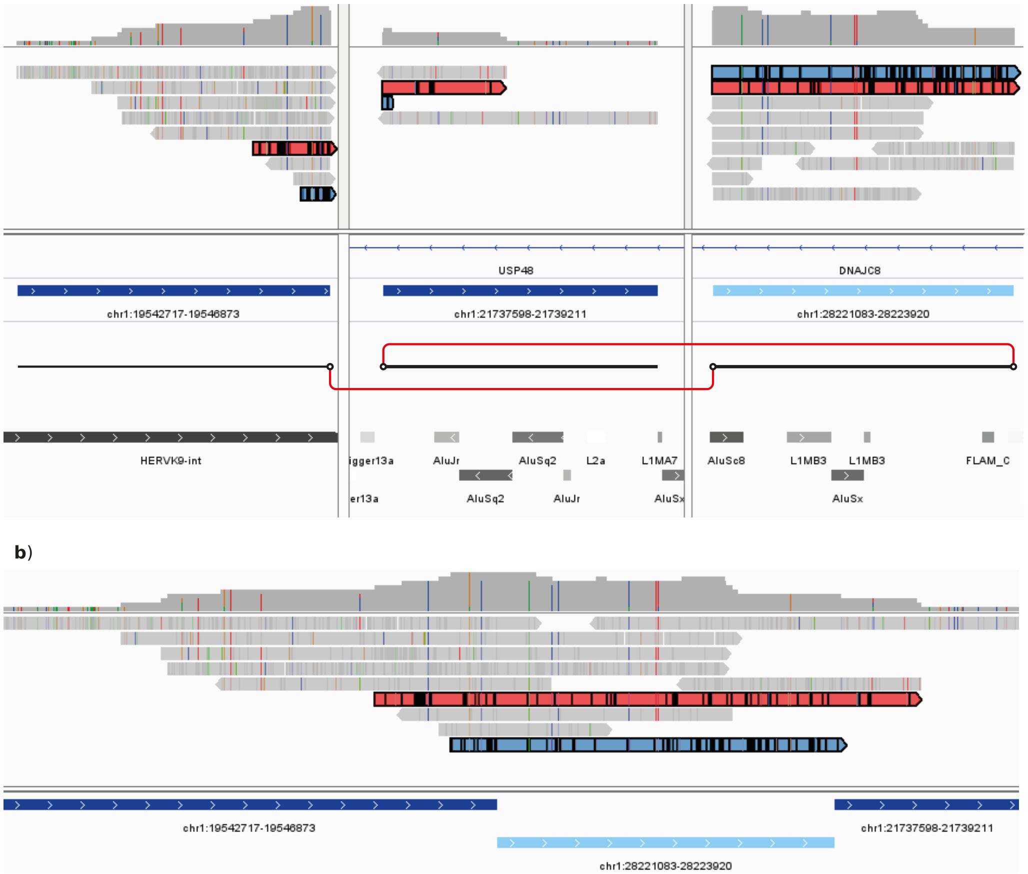

Figure S11: Supporting evidence for the 2.2 Mb deletion with additional 2.8 kb insertion on chr1. Reads supporting the junction from chr1:19.546.873-chr1:28.221.084 and chr1:28223920-chr1:21737599 were extracted and mapped to (a) GRCh38 and (b) a reference containing the genomic regions adjacent the SV breakpoints. Track annotation shows the exact genomic regions. The light blue annotation indicates the 2.8 kb insertion. Reads supporting both SVs are highlighted in red and blue. The junctions are shown in red connecting the black genomic segments. Repeats located around the genomic segments are extracted from UCSC genome browser RepeatMasker track and grayscaled by divergence with lighter color indicating higher divergence.

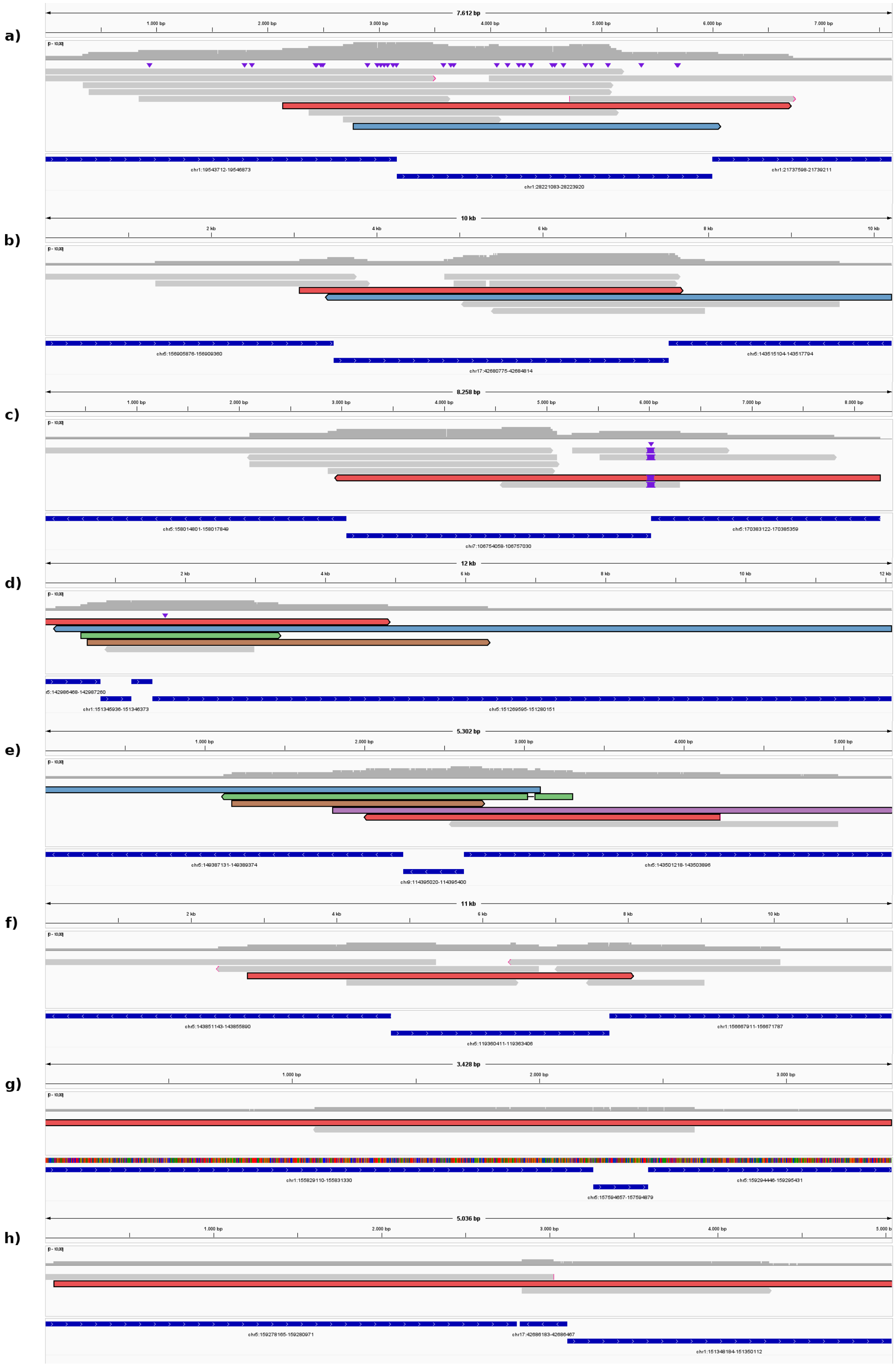

Figure S12: Templated insertion SV with supporting reads. The assembled regions of eight templated insertion SVs (including the flanking and inserted regions) are shown as one contig. Supporting reads are shown below and reads spanning the complete templated insertion are marked.

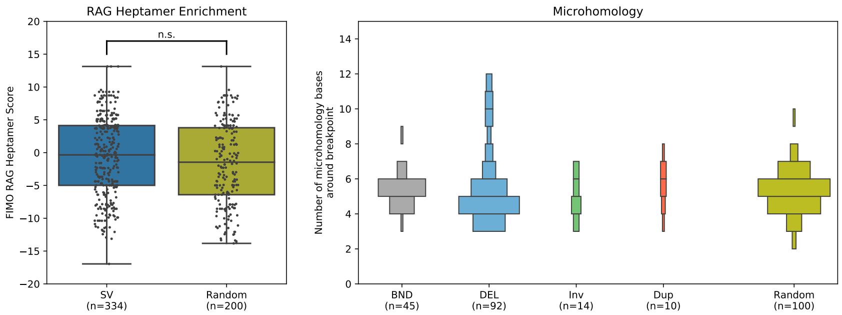

Figure S13: No enrichment of RAG Heptamers (a) or microhomology (b) at SV breakpoints. (a) At the breakpoints of the SVs as well as 100 randomly chosen SVs (Random), RAG heptamer score was calculated as described in Choi et al. 2015. (b) Microhomology stretches for SVs and 100 randomly chose SVs were calculated. The SV are divided by SV type. The high scoring deletions (more than 10 bases of microhomology) are in 6 of 7 cases 6 kb deletions of reference genome L1HS LINE elements.

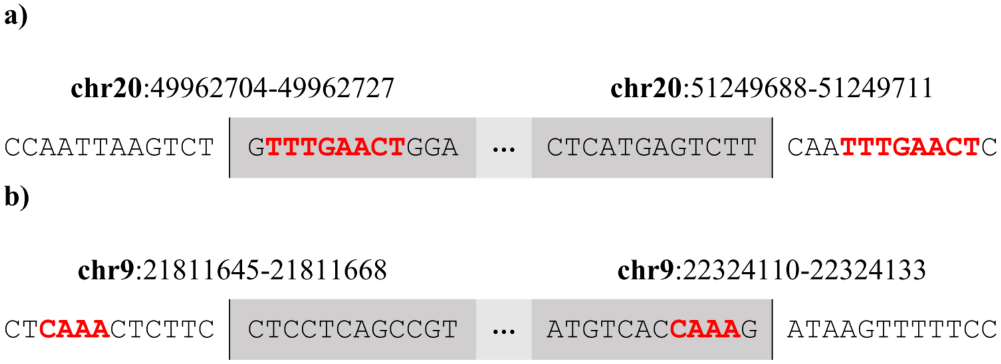

Figure S14: Putative 8 bp microhomology on the flanks of a deletion on chr20 (a) and best match for homology detection in the *CDKN2A* affecting deletion on chr9 (b). The homologous bases are colored red, the genomic region deleted by the deletion are highlighted in gray. The breakpoint is marked by the black line.

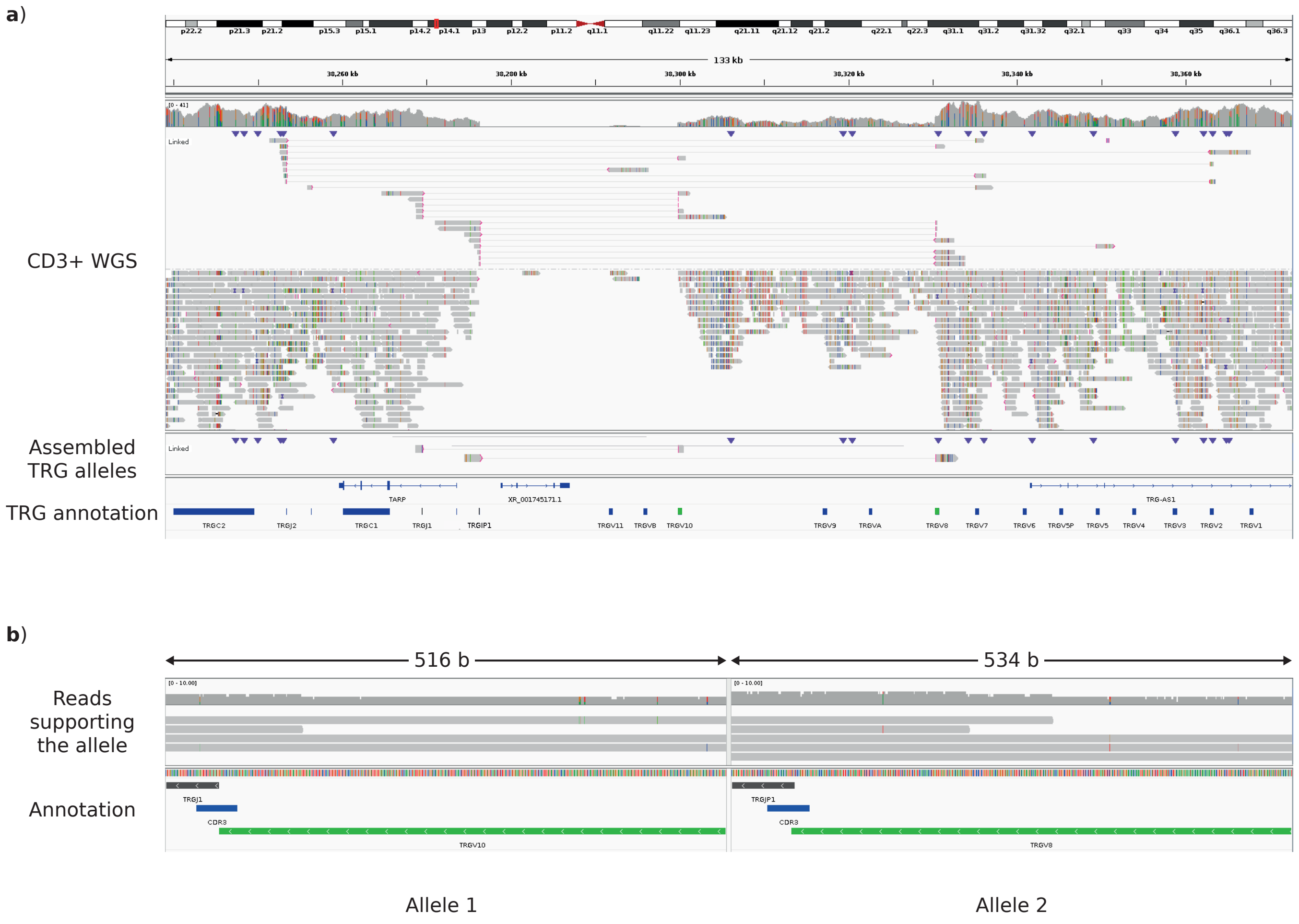

Figure S15: Rearrangement of T-cell receptor gamma. (a) Shown is the sequencing data at the T-cell receptor gamma locus. Multiple reads with primary and supplementary alignment at V- and J-segments indicate rearranged T-cell receptor alleles. The most common V/J combinations, TRGV10-TRGJ1 and TRGV8-TRGJP1, were assembled. (b) Detailed view on the V/J junction and CDR3 region of both assembled alleles.

2 Supplementary Tables

Table S1: Primers used for amplification of selected breakpoints and subsequent Sanger sequencing.

**Primer**	**Sequence**	**Target**
chr9_DEL_F<br>chr9_DEL_R	TCAGACCCTTGGATTGATTTGC<br>TGTCAGTATGCACCCCTGATT	chr9:21.8-22.3 Mb deletion
chr9_INV_long_F<br>chr9_INV_long_R	TACAAGTGTGGCTGCTCTGG<br>TGGAGGACTTCAGATGGTGA	chr9:2.8-34.4 Mb inversion
chr9_INV_short_F<br>chr9_INV_short_R	CTGCAGCTTGTGAGGTCCTT<br>TGCGGAGGGTGTACTGAGT	chr9:23.8-34.5 Mb inversion
chr9-chr17_BND_F<br>chr9-chr17_BND_R	GCCCTCAAGCCTCTCAGATG<br>CAGGCCCTCGACTGAAAAGT	chr9:134.1 Mb - chr17:42.6 Mb translocation
chr16-chr17_BND_F<br>chr16-chr17_BND_F	GCGACAACTGAACAGTGAGC<br>TGACCCTGGAGCCTAACTGT	chr16:70.3 Mb - chr17:42.1 Mb translocation

Table S2: Regions targeted by adaptive sampling Oxford Nanopore sequencing.

Chromosome	Start	End	Length
chr1	16665842	16695842	30000
chr1	18991193	28861526	9870333
chr1	42643197	42673197	30000
chr1	43067063	43097063	30000
chr1	146037341	146067341	30000
chr1	146139856	146169856	30000
chr1	149348971	149378971	30000
chr1	150936619	150966619	30000
chr1	151330938	151361373	30435
chr1	153693771	153728605	34834
chr1	155710056	155716793	6737
chr1	155827427	155853084	25657
chr1	156652912	156682912	30000
chr1	184072101	184477768	405667
chr1	186155923	186184402	28479
chr1	228152397	228182397	30000
chr1	247646312	247676312	30000
chr1	248505422	248535422	30000
chr2	32702104	32732104	30000
chr2	151020613	151050613	30000
chr2	200487333	200517333	30000
chr2	201266646	201296646	30000
chr2	204763567	204793567	30000
chr3	8690438	8720439	30001
chr3	9930682	9960682	30000
chr3	130736789	130766791	30002
chr3	161775742	161805742	30000
chr4	64149232	64179232	30000
chr4	64532046	64562046	30000
chr4	80335952	80363345	27393
chr4	83011572	83035089	23517
chr4	175426786	175456786	30000
chr5	3647217	3674599	27382
chr5	115014729	172576410	57561681
chr6	3030232	3060232	30000
chr6	16439214	16469214	30000
chr6	29592911	29622911	30000
chr6	31502586	31532586	30000
chr6	98800393	98830393	30000
chr6	106791522	106821551	30029
chr6	108083876	108113876	30000
chr6	108281809	108311809	30000
chr6	109729690	109759692	30002
chr6	160943870	160973870	30000

Chromosome	Start	End	Length
chr7	18401115	18431115	30000
chr7	38232695	38375133	142438
chr7	67007287	67037287	30000
chr7	72790252	72820252	30000
chr7	106739059	106769059	30000
chr7	107373004	107412234	39230
chr7	108948340	108978340	30000
chr8	125510423	125540431	30008
chr9	1	40000000	39999999
chr9	114277635	114307642	30007
chr9	114594667	114624667	30000
chr9	129755834	129785834	30000
chr9	130623887	130668384	44497
chr9	134012510	134220856	208346
chr10	1034798	1064798	30000
chr10	14359811	14389811	30000
chr10	101498337	101528337	30000
chr10	103356258	103390167	33909
chr10	114442896	114472896	30000
chr10	114889206	114919206	30000
chr11	63825100	63855100	30000
chr11	63922915	63952915	30000
chr11	67232007	67240324	8317
chr11	67276971	67306971	30000
chr11	102329433	102359433	30000
chr11	117126906	117156906	30000
chr12	6598870	6629716	30846
chr12	55914777	55944777	30000
chr12	56044195	56080287	36092
chr13	49602382	49632382	30000
chr13	51311407	51341407	30000
chr14	31342649	31372649	30000
chr14	32555265	32602883	47618
chr14	33819618	33849741	30123
chr14	35003452	35033452	30000
chr14	36689392	36719392	30000
chr14	73514152	73544152	30000
chr14	73545393	73575393	30000
chr14	73654586	73684586	30000
chr14	73943045	73973046	30001
chr15	84746420	84776420	30000
chr16	21568123	21598123	30000
chr16	22684431	22714431	30000
chr16	68546804	68576804	30000
chr16	68672971	68702971	30000
chr16	68964377	68994377	30000
chr16	69793638	69823638	30000
chr16	69851839	69881839	30000
chr16	70339297	70396800	57503

**Chromosome**	**Start**	**End**	**Length**
chr17	41999900	42757169	757269
chr17	43478020	43508020	30000
chr18	21461725	21491725	30000
chr18	21577615	21607615	30000
chr19	1047549	1077549	30000
chr19	1257556	1270847	13291
chr19	1835065	1865065	30000
chr19	40555682	40585682	30000
chr19	40593967	40623967	30000
chr19	46818477	46848477	30000
chr20	31834755	31874134	39379
chr20	35459387	35489387	30000
chr20	49947714	49977714	30000
chr20	51234700	51264700	30000
chr20	54342457	54372457	30000
chr20	55285771	55315771	30000
chr22	28649069	28685108	36039
chr22	42490533	42520533	30000
chr22	42543361	42573361	30000
